# Glycosidic α-linked mannopyranose disaccharides: an NMR spectroscopy and molecular dynamics simulation study employing additive and Drude polarizable force fields†

**DOI:** 10.1039/d2cp05203b

**Published:** 2022-12-21

**Authors:** Alessandro Ruda, Asaminew H. Aytenfisu, Thibault Angles d’Ortoli, Alexander D. MacKerell, Göran Widmalm

**Affiliations:** a Department of Organic Chemistry, Arrhenius Laboratory, Stockholm University S-106 91 Stockholm Sweden goran.widmalm@su.se; b Department of Pharmaceutical Sciences, School of Pharmacy, University of Maryland Baltimore Maryland 21201 USA alex@outerbanks.umaryland.edu

## Abstract

d-Mannose is a structural component in N-linked glycoproteins from viruses and mammals as well as in polysaccharides from fungi and bacteria. Structural components often consist of d-Man*p* residues joined *via* α-(1→2)-, α-(1→3)-, α-(1→4)- or α-(1→6)-linkages. As models for these oligo- and polysaccharides, a series of mannose-containing disaccharides have been investigated with respect to conformation and dynamics. Translational diffusion NMR experiments were performed to deduce rotational correlation times for the molecules, 1D ^1^H,^1^H-NOESY and 1D ^1^H,^1^H-T-ROESY NMR experiments were carried out to obtain inter-residue proton–proton distances and one-dimensional long-range and 2D J-HMBC experiments were acquired to gain information about conformationally dependent heteronuclear coupling constants across glycosidic linkages. To attain further spectroscopic data, the doubly ^13^C-isotope labeled α-d-[1,2-^13^C_2_]Man*p*-(1→4)-α-d-Man*p*-OMe was synthesized thereby facilitating conformational analysis based on ^13^C,^13^C coupling constants as interpreted by Karplus-type relationships. Molecular dynamics simulations were carried out for the disaccharides with explicit water as solvent using the additive CHARMM36 and Drude polarizable force fields for carbohydrates, where the latter showed broader population distributions. Both simulations sampled conformational space in such a way that inter-glycosidic proton–proton distances were very well described whereas in some cases deviations were observed between calculated conformationally dependent NMR scalar coupling constants and those determined from experiment, with closely similar root-mean-square differences for the two force fields. However, analyses of dipole moments and radial distribution functions with water of the hydroxyl groups indicate differences in the underlying physical forces dictating the wider conformational sampling with the Drude polarizable *versus* additive C36 force field and indicate the improved utility of the Drude polarizable model in investigating complex carbohydrates.

## Abstract 2

A wider conformational sampling takes place with the CHARMM Drude polarizable *versus* the additive C36 force field.

## Graphical abstract

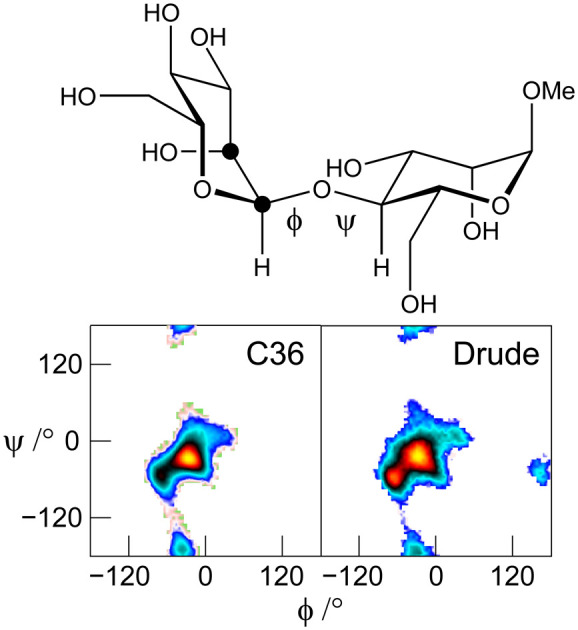

## Introduction

Complex carbohydrates, also referred to as glycans, are essential components in biological systems.^1,2^ These glycans show a multitude of diverse roles in biology and medicine^3^ such as facilitating physical structure, protection against proteases and pathogens, intracellular signaling and adhesion as well as being components of antigen^4^ or bacteriophage^5^ recognition, immune modulation, and antigenic epitopes formed on cell surfaces. Structurally the glycans are found as glycoconjugates including glycoproteins,^6^ glycolipids,^7^ lipoglycans^8^ and saponins,^9^ or as reducing oligosaccharides, in particular milk oligosaccharides from mammals.^10^ Due to their importance in biological processes, development of glycomimetics, which mimic structure and function of native carbohydrates with the aim of blocking specific carbohydrate-protein interactions, has been pursued in the area of medicinal chemistry.^11,12^

d-Mannose^13^ is a common monosaccharide in nature. It is a structural component in N-linked glycans in the form of high-mannose, hybrid or complex types as part of multi-branched structures.^14^ In virus glycoproteins the extent of under-processed oligomannose-type glycans differ depending on the site of glycosylation, and are found as Man_9_GlcNAc_2_ to Man_5_GlcNAc_2_ structures. Different substitution patterns are observed on the protein backbone where the glycans act as shields on, for example, Lassa,^15^ HIV,^16–18^ an α-coronavirus^19^ and SARS-CoV-2^20,21^ viruses. Besides the β-linked branching core mannose residue of common N-linked glycoproteins mannose residues are α-(1→2)-, α-(1→3)-, and α-(1→6)-linkages to a mannose residue in these structures. In fungi there are glycoproteins containing cores with extended polymeric α-(1→6)-linked mannoses having short α-(1→2)-linked mannose chains^22^ capped with α-(1→3)-linked mannose structures^23^ and in *Candida albicans* yeast mannans α-d-Man*p*-(1→2)-α-d-Man*p* and α-d-Man*p*-(1→3)-α-d-Man*p* can be found as structural elements.^24^ In the filamentous fungus *Aspergillus fumigatus* the major cell wall molecule is a galactomannan with a linear mannan having α-(1→6)- and α-(1→2)-linked mannose residues; these mannans are an integral part of the cell wall where they are covalently bound to the glucan-chitin core.^25^ Mannose-containing oligosaccharide structures that have α-(1→2)-linkages in lipoarabinomannans from *Mycobacterium tuberculosis*^26^ are required for the C-lectin Dectin-2 to mediate binding and recognition.^27^ The fimbrial lection FimH from uro- and enteropathogenic *Escherichia coli* bacteria binds efficiently to oligomannose glycans that are α-(1→3)-linked at their non-reducing end.^28^ Polymethylated polysaccharides from *Streptomyces griseus*^29^ and *M. smegmatis*^30^ are composed of α-(1→4)-linked 3-*O*-methyl-d-mannose residues, which in the latter bacterial polysaccharide is capped by a single non-methylated α-(1→4)-linked mannose residue. Interestingly, the reducing end mannosyl residue of the α-(1→4)-linked 3-*O*-methyl-d-mannose polysaccharide from *Mycobacterium hassiacum* as well as from other nontuberculous mycobacteria is present as a methyl glycoside formed by the action of a unique *S*-adenosyl-l-methionine-dependent sugar 1-*O*-methyltransferase.^31^ MeT1 from *M. hassiacum* was active on the di-*O*-methylated α-d-Man*p*3Me-(1→4)-d-Man*p*3Me as a substrate but not on α-d-Man*p*-(1→4)-d-Man*p* devoid of 3-*O*-methyl groups. Thus, as part of model compounds α-d-Man*p*-OMe residues at the reducing end are not only suitable in order to avoid the equilibrium mixture resulting from different anomeric configurations but this capping is actually present in nature. Hence, mannose in the pyranose ring form, which has four hydroxyl groups available for substitution (the anomeric position exempt), has in nature been found to be glycosylated by α-linked mannose at one or more of these positions.

**Fig. 1:**
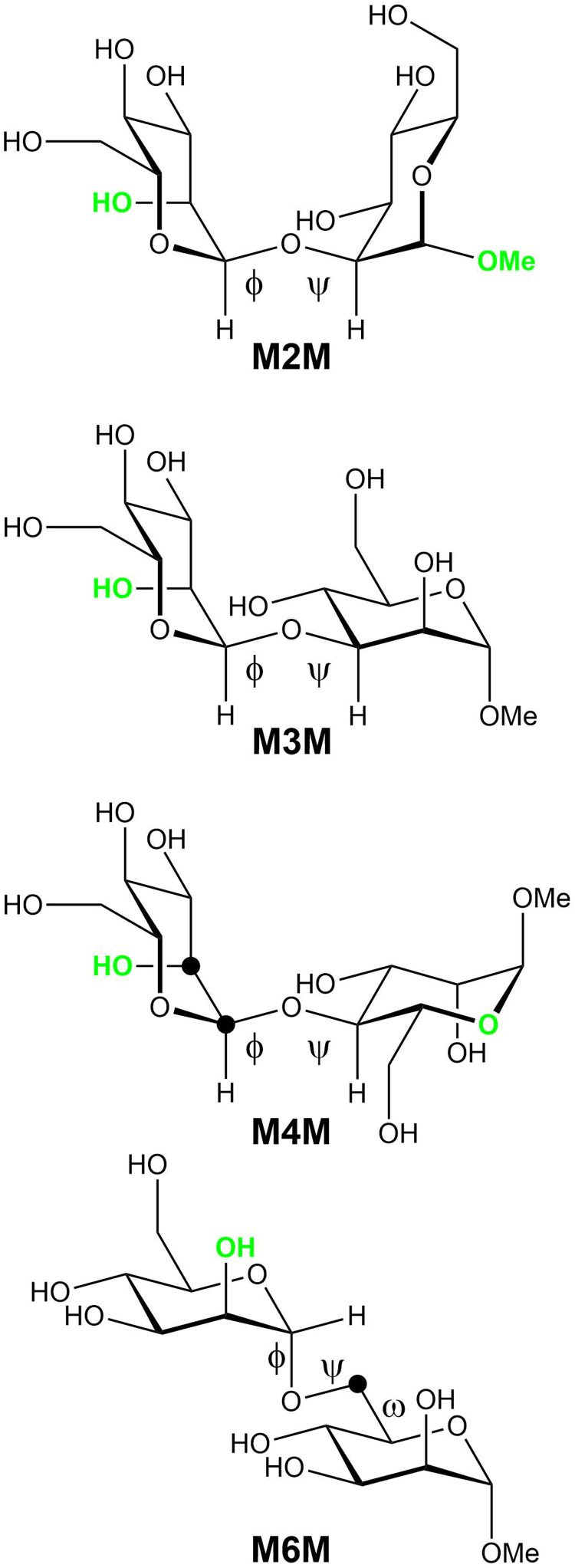
Schematic of four glycosidically α-linked mannopyranose disaccharides with glycosidic torsion angles *ϕ* and *ψ* as well as the *exo*-cyclic torsion angle *ω* identified. Atomic positions specifically ^13^C-labeled in different versions of some compounds are highlighted by filled black circles; these compounds are then referred to as M4M-*c*_2_ and M6M-*c*. Electronegative hydroxyl groups or oxygen atoms oriented such that a constant in-plane (CIP) effect operates, which results in an enhanced ^3^*J*_CC_ along the coupling pathway, are shown in green color.

To study the conformations and dynamics of oligosaccharides in solutions the combined use of NMR spectroscopy and molecular simulations offers a very powerful approach,^32^*e.g.*, of high-mannose type oligosaccharides.^33^ Herein we extend our previous studies on mannose-containing oligosaccharides that utilized energy-resolved mass spectrometry,^34^ which revealed the order of stability of mannosyl linkages as follows: 6-linked > 4-linked ≧ 2-linked > 3-linked mannosyl residues, and Raman optical activity^35^ (ROA) for which a full quantum mechanical/molecular mechanical approach was required for an optimal calculation of the ROA parameters. The current study aims to extend the acquired knowledge herein focusing on the conformation and dynamics of the four α-(1→2)-, α-(1→3)-, α-(1→4)-, and α-(1→6)-linked mannose disaccharides as α-linked methyl glycosides (Fig. 1) by NMR spectroscopy and molecular dynamics simulations using both additive CHARMM36 and Drude polarizable force fields.

## Experimental section

### General

Disaccharides available from previous studies were α-d-Man*p*-(1→2)-α-d-Man*p*-OMe (M2M) and α-d-Man*p*-(1→3)-α-d-Man*p*-OMe (M3M),^36^ α-d-Man*p*-(1→4)-α-d-Man*p*-OMe (M4M),^34^ α-d-Man*p*-(1→6)-α-d-Man*p*-OMe (M6M) and α-d-Man*p*-(1→6)-α-d-[6-^13^C]Man*p*-OMe (M6M-*c*).^37^

### Synthesis

The synthesis of α-d-[1,2-^13^C_2_]Man*p*-(1→4)-α-d-Man*p*-OMe (M4M-*c*_2_) will be briefly described using d-[1,2-^13^C_2_]mannose with a 99 atom-% enrichment (Omicron Biochemicals, Inc., South Bend, IN, USA). The donor (2-methyl-5-*tert*-butylphenyl) 2,3,4,6-tetra-*O*-acetyl-1-thio-α-d-[1,2-^13^C_2_]mannopyranoside was prepared in a similar way to its per-*O*-benzoylated analogue^38^ and the acceptor methyl 2,3,6-tri-*O*-benzyl-α-d-mannopyranoside,^34^ was made by regioselective reductive ring opening of the benzylidene acetal in methyl 2,3-di-*O*-benzyl-4,6-benzylidene-α-d-mannopyranoside using triethylsilane and iodine in acetonitrile.^39^ The donor and acceptor molecules were coupled in an *N*-iodosuccinimide/silver triflate promoted glycosylation reaction.^40,41^ Standard workup, silica-based purification, and deprotection *via* a two-step procedure using (i) NaOMe in MeOH and (ii) triethylsilane and Pd/C in MeOH^42^ yielded the deprotected disaccharide α-d-[1,2-^13^C_2_]Man*p*-(1→4)-α-d-Man*p*-OMe, which was purified by gel-permeation chromatography. ^1^H and ^13^C NMR chemical shift data in agreement with those previously reported;^34^^1^*J*_C1′,C2′_ = 47.4 Hz. HR-ESI-MS: *m/z* calc. for ^13^C_2_C_11_H_24_NaO_11_ 381.1278, found 381.1290.

### NMR spectroscopy

Disaccharides were dissolved in D_2_O and M3M and M4M samples (pD ≈ 6) were prepared in 5 mm NMR tubes with a sample concentration of 56–62 mM; M6M and M6M-*c* were prepared at a sample concentration of 61 and 56 mM, respectively, whereas M2M had a concentration of 4 mM. Disaccharide M4M-*c*_2_ was prepared in a 4 mm NMR tube to a concentration of 91 mM. ^1^H chemical shifts were referenced to internal sodium 3-trimethylsilyl-(2,2,3,3-^2^H_4_)-propanoate (TSP, *δ*_H_ 0.00). NMR experiments were carried out at 310 K on a 500 MHz Bruker AVANCE spectrometer equipped with a 5 mm TCI Z-gradient Cryoprobe or at 298 K or 310 K on a 600 MHz Bruker AVANCE III spectrometer equipped with a 5 mm inverse Z-gradient TXI (^1^H/^13^C/^31^P) or with a 5 mm BBO probe as well as on a 700 MHz Bruker AVANCE III equipped with a 5 mm TCI Z-Gradient Cryoprobe (^1^H/^13^C/^15^N). The temperature was calibrated by a methanol-*d*_4_ sample,^43^ and processing of the acquired data was carried out using TopSpin 3.1 and 4.0.1, Dynamic Center 2.7.2 (Bruker Daltonics) and in-house processing scripts. The translational diffusion experiment was repeated 3 to 13 times and the results reported as arithmetic averages. A pulsed-field-gradient spin-echo experiment^44^ with a fixed diffusion time (*Δ* = 50 or 100 ms) and a pulsed-field-gradient increasing linearly over 32 steps was used, ranging from 2% to 95% of the maximum gradient strength being 55.7 G s^−1^. The pulsed-field-gradients were calibrated using a doped water sample (1% H_2_O in D_2_O containing 1 mg mL^−1^ GdCl_3_) and a literature value for the translational diffusion coefficient *D*_t_ of 1.90 × 10^−9^ m^2^ s^−1^ for the HDO diffusion coefficient in D_2_O at 25 °C.^45^

^1^H,^1^H cross-relaxation rates were determined at 310 K employing 1D ^1^H,^1^H-NOESY^46^ and 1D ^1^H,^1^H-T-ROESY^47^ experiments at a ^1^H frequency of 600 MHz except for the 1D ^1^H,^1^H-T-ROESY experiment on M6M-*c* which was carried out at 298 K and a ^1^H frequency of 500 MHz. In these cases, selective excitation was achieved by single or double PFGSE modules utilizing 30–120 ms r-SNOB shaped pulses for the NOESY experiment and 50 ms r-SNOB shaped pulses for the T-ROESY experiment. The strengths of the first and second gradient pairs were 15% and 40%, respectively, of the maximum (55.7 G cm^−1^) for the NOESY experiments. For the T-ROESY, the strengths of the gradients were set to 5.7% or 12.5%. In the NOESY experiments, zero-quantum coherences were suppressed using the scheme devised by Thrippleton and Keeler^48^ where a 20 ms adiabatic Chirp pulse with a bandwidth of 20–40 kHz was applied together with a gradient pulse with 6–7% of the maximum power. The T-ROESY spinlock was applied with *γB*_1_/2π = 2.5 kHz. For the NOESY as well as the T-ROESY experiments, 10–14 cross-relaxation delays between 50–600 ms were collected for each of the excited spins. A spectral width of 6 ppm was sampled using 16k data points and 512 transients were averaged. The repetition time was 8–10 s, *i.e.*, in all cases longer than 5 × *T*_1_. Prior to Fourier transformation, the FIDs of the 1D experiments were zero-filled to 256k points and multiplied by an exponential line-broadening function of 2 Hz. Baseline correction was performed prior to integration, which used the same integration limits for all experiments within a series. The areas of relevant peaks were divided by the area of the inverted peak and least-square fitted to a first order function yielding the cross-relaxation rate constant.

For the M4M sample, measurements of the transglycosidic carbon–proton coupling constants were performed using *J*-HMBC experiments^49^ and one-dimensional long-range (1DLR) experiments essentially as devised by Nishida *et al.*^50,51^ For the *J*-HMBC experiments, a threefold low-pass *J*-filter (*J* = 140 Hz, 155 Hz and 175 Hz) was used to suppress ^1^*J*_CH_. Scaling factors ranging approximately from 34 to 65, calculated from *κ* = Δ/*t*^max^_1_, where Δ was at least 60% of the inverse of the smallest coupling constant to be measured, were used to scale the coupling in the indirect (*F*_1_) dimension. A spectral width of 6 ppm for ^1^H and 175 ppm for ^13^C were used. The experiments were performed with 16 384 × 512 points and 32 scans per *t*_1_ increment with the echo/antiecho method. Forward linear prediction to 1024 points in the *F*_1_ dimension and subsequent zero-filling to 8192 points was applied prior to Fourier transformation. Coupling constants were extracted from 1D-projections of the resonances of interest. One-dimensional long-range (1DLR) experiments employed ^13^C site-selective excitation with a Gaussian shaped pulse of 160 ms duration. The delay used for suppression of ^1^*J*_C,H_ was set to (145 Hz)^−1^ and the time of the delay between excitation and coherence transfer, for evolution of the long-range coupling, was set by using a nominal value of 8–16 Hz; an acquisition time of 3 s, 1024–2048 transients and 50k data points were used. Zero-filling was performed to 512k data points and an exponential line-broadening function lb = 0.6 Hz was used. Subsequently, the ^3^*J*_CH_ coupling constants were extracted by the *J* doubling methodology^52^ implemented in-house by a MATLAB script.

For M4M-*c*_2_ multiple-bond homonuclear ^13^C,^13^C coupling constants were obtained at 333 K from a ^13^C{^1^H} NMR spectrum at 175 MHz using an acquisition time of 4 s and 128k data points. The FID was zero-filled to 512k points and a Lorentz-Gaussian window function (lb = −1.0, gb = 0.5) was applied followed by deconvolution using a Lorentzian line-shape fitting (TopSpin 4.0.1) to obtain ^*n*^*J*_CC_. The transglycosidic long-range heteronuclear ^1^H,^13^C coupling constant related to the torsion angle ϕ was measured by ^13^C-J-HMBC experiments using a constant-time version to suppress ^13^C,^13^C scalar couplings.^53^ A threefold low-pass *J*-filter (*J* = 145 Hz, 160 Hz and 180 Hz) was used to suppress ^1^*J*_CH_. Scaling factors *κ* of 18 and 33 were employed. Spectral widths of 10 ppm and 60 ppm were used for the ^1^H and ^13^C dimensions, respectively. Each experiment had 64 scans per *t*_1_ increment, a FID size of 4096 × 512 number of points and was prior Fourier transform zero-filled to 8192 × 2048 points in the *F*_2_ and *F*_1_ dimensions, respectively. Peak separation along the *F*_1_ dimension was extracted from 1D-projections of the resonances of interest and the coupling constant values were calculated by *J*_CH_ = Δ*F*_1_/*κ*, with Δ*F*_1_ being the measured peak separation. The reported transglycosidic ^3^*J*_H1′,C4_ coupling constant is the average value of the two measurements. A ^1^H,^13^C-HSQC-HECADE NMR experiment^54,55^ was applied to M4M-*c*_2_ at 293 K to determine the sign and magnitude of ^2^*J*_C2′,H1′_. The DIPSI-2 isotropic mixing scheme was applied with a duration of 50 ms and a *t*_1_*/*t*_1_ scaling factor of unity was used. The experiment was recorded with 16 scans per *t*_1_ increment and a relaxation delay of 2 s. The 2D data matrix contained 16 384 × 256 data points in the *F*_2_ and *F*_1_ dimensions, respectively, covering spectral widths of 4 ppm and 100 ppm in the ^1^H and ^13^C dimensions. Prior to Fourier transformation zero-filling was carried out to 2k data points in the *F*_1_ dimension and 90° shifted squared sine-bell functions were applied in both dimensions.

### *J*-Based conformational analysis

From the MD simulations of the four mannobioses one-, two- and three-bond spin–spin NMR coupling constants were calculated utilizing the following equations:

1 ^1^*J*_C1′,H1′_(*ϕ*_H_) = 1.32 cos(2*ϕ*_H_) − 3.38 cos(*ϕ*_H_) − 1.05 sin(2*ϕ*_H_) + 1.27 sin(*ϕ*_H_) + 168.9 + 0.0390 (ε)

2 ^2^*J*_C2′,H1′_(*ϕ*_H_) = −2.26 + 1.61 cos(*ϕ*_H_) − 0.89 cos(2*ϕ*_H_) + 0.59 sin(*ϕ*_H_) + 0.93 sin(2*ϕ*_H_)

3 ^2^*J*_C1′,C2_(*ϕ*_H_) = −2.56 + 0.93 cos(*ϕ*_H_) + 0.34 sin(*ϕ*_H_) − 0.35 cos(2*ϕ*_H_) + 0.42 sin(2*ϕ*_H_)

4 ^2^*J*_C1′,C*n*_(*ϕ*_H_) = −2.79 + 1.15 cos(*ϕ*_H_) + 0.10 sin(*ϕ*_H_) − 0.17 cos(2*ϕ*_H_) + 0.47 sin(2*ϕ*_H_)

5 ^3^*J*_H1′,C*n*_(*ϕ*_H_) = 6.54 cos^2^(*ϕ*_H_ − Δ − *Θ*) − 0.62 cos(*ϕ*_H_ − Δ − *Θ*) − 0.17

6 ^3^*J*_C1′,H*n*_(*ψ*_H_) = 6.54 cos^2^(*ψ*_H_) − 0.62 cos(*ψ*_H_) + 0.33 + 0.6exp(*κ *cos(*ϕ*_O5′_ – 180))/exp(*κ*)

7 ^3^*J*_C2′,C*n*_(*ϕ*_C2′_) = 3.72 cos^2^(*ϕ*_C2′_ + Δ) − 0.08 + CIP

8 ^3^*J*_C1′,C*n*±1_(*ψ*_C*n*±1_) = 4.28 cos^2^(*ψ*_C*n*±1_) − 0.11 + 0.6exp(*κ* cos(*ϕ*_O5′_ – 180))/exp(*κ*) + CIP

9 ^3^*J*_H5,H6pro-*R*_(*ω*) = 5.08 + 0.47 cos(*ω*) + 0.90 sin(*ω*) − 0.12 cos(2*ω*) + 4.86 sin(2*ω*)

10 ^3^*J*_H5,H6pro-*S*_(*ω*) = 4.92 − 1.29 cos(*ω*) + 0.05 sin(*ω*) + 4.58 cos(2*ω*) + 0.07 sin(2*ω*)

11 ^3^*J*_H5,C6_(*ω*) = −1.29 + 1.53 cos(*ω*) − 3.68 sin(*ω*)

Glycosidic torsion angles in the mannobioses are represented by *ϕ* and *ψ* where atoms in the terminal sugar residue are primed. Torsion angles are given by *ϕ*_H_ = H1′–C1′–O*n*–C*n*, *ϕ*_C2′_ = C2′–C1′–O*n*–C*n*, *ψ*_H_ = C1′–O*n*–C*n*–Hn, *ψ*_C*n*±1_ = C1′–O*n*–C*n*–C(n ± 1), where n is the linkage position. The exocyclic hydroxymethyl torsions are defined by *ω* = O5–C5–C6–O6 and *ω*′ = O5′–C5′–C6′–O6′.

A conformational dependence of the anomeric ^1^*J*_CH_ coupling constant on the glycosidic torsion angle *ϕ* has been reported by Tvaroska and Taravel,^56^ where *ε* is the dielectric permittivity assumed for water solution as *ε* = 80 (eqn 1), and a heteronuclear two-bond coupling constant relationship was recently described by Serianni and co-workers for the torsion angle *ϕ* (eqn 2).^57^ Transglycosidic two-bond ^13^C,^13^C-homonuclear coupling constants employ recently developed equations for α-linked mannobioses, 2-linked (eqn 3), 3-linked (eqn 4);^58^ for the 4-linked mannobiose M4M eqn 4 was employed. Heteronuclear (eqns 5 and 6) and homonuclear (eqn 7 and 8) transglycosidic Karplus-type relationships were employed, where a phase shift (Δ), being dependent on the anomeric and absolute configuration, is set to –12° for the heteronuclear three-bond *ϕ* glycosidic torsion angles, since the terminal residues are α-d-hexopyranosides;^59^ Θ is and additional phase shift further added to the originally proposed equation and is set as +6° (eqn 5).^32^ The variable in-plane (VIP) effect is implemented by setting *κ* = 8. The constant in-plane (CIP) effect of 0.6 Hz for the mannosyl residues is present in the ^13^C,^13^C-coupling pathway for all mannobioses due to the electronegative^60,61^ axially oriented HO2′ group related to *ϕ* as well as OMe in M2M and O5 in M4M related to *ψ*, as highlighted in Fig. 1. The in-plane effect results in an increased coupling between C and Z for the fragment O–C–X–Y–Z when the dihedral angle O–C–X–Y is antiperiplanar, *i.e.*, when the terminal oxygen atom in the sequence lies in the C–X–Y plane. The glycosidic torsion angle of the reducing end residue is denoted by *ϕ*_Me_ = H1–C1–O1–C(Me).

For the *ω* torsion angle in M6M defined by O5–C5–C6–O6 the calculations from the MD simulation of the ^3^*J*_H5,H6pro-*R*_ and ^3^*J*_H5,H6pro-*S*_ coupling constants employed the Karplus relationships given by Stenutz *et al.*^62^ and for the ^2^*J*_H5,C6_ coupling constant the relationship given by Thibaudeau *et al.*^63^ was used.

### Computational details

Molecular dynamics (MD) simulations of the four α-linked mannopyranose disaccharides were performed with the program CHARMM^64–66^ and NAMD^67,68^ under the additive CHARMM36^69–71^ and polarizable Drude force fields for carbohydrates^72^ with the CHARMM TIP3P water model^73,74^ for the additive force field and the SWM4-NDP water model^75^ for the Drude force field. The time-steps were 2 fs and 1 fs in the MD simulations when using the additive and polarizable Drude force fields, respectively. Each MD simulation contained four disaccharides solvated in a 32 Å × 32 Å × 32 Å water box to maintain 200 mM solutions.^70^ All simulations were performed under the *NPT* ensemble with the temperature maintained at 298 K using the Hoover algorithm with a thermal piston mass of 1000 kcal mol^−1^ ps^−2^ and using the Langevin piston algorithm with a collision frequency of 20 ps^−1^ and mass of 1630 amu to maintain the pressure^76^ for the additive simulations with CHARMM. For the Drude simulations with NAMD the extended Lagrangian approach with a dual-Langevin thermostat was used for integrating the equations of motion, where the temperature was maintained at 298 K for real atoms and at 1 K for Drude oscillators with thermostat friction coefficients of 5 ps^−1^ and 20 ps^−1^, respectively,^68^ with the pressure maintained using the Langevin algorithm with a piston oscillation period of 200 fs and a relaxation time of 100 fs. SHAKE was used to constrain covalent bonds involving hydrogen atoms.^77^ Short-range LJ forces were force switched (additive) or potential switched (Drude) to zero from 10–12 Å.^78^ Electrostatic interactions were computed with the smooth particle mesh Ewald (PME) method with a real space cutoff of 12 Å, a *κ* factor of 0.34 and a 6-order spline.^79^ The Drude Hardwall constraint was set at 0.2 Å to prevent polarization catastrophe^80^ and analysis was undertaken to assure that the nuclei-Drude particle distances were not reaching 0.2 Å.

For the additive force field conformational sampling was enhanced by applying the Hamiltonian replica exchange with concurrent solute scaling and biasing potential (HREST2-BP) method.^81,82^ In HREST-BP simulations, the scaling temperatures were assigned to 303 K, 324 K, 351 K, 382 K, 414 K and 450 K, with the ground-state replica temperature of 303 K selected to correspond to the experimental temperature range.^32^ Each replica was simulated for 100 ns with coordinates saved every 10 ps for analysis that was based on the ground-state replica. The Hamiltonian biasing potential was constructed using the 2-dimensional (2D) grid-based correction map (bpCMAP) along the torsions O5′–C1′–O*n*–C*n*/C1–O*n*–C*n*–C*n* + 1 for M2M, M3M and M4M where O*n*, C*n* and C*n* + 1 are atoms in the reducing-end monosaccharide of the linkage.^32^ For M6M a combination of the 2D bpCMAP and a Saxon−Woods potential^83^ were used with the bpCMAP applied to the O5–C1–O6–C6/C1–O6–C6–C5 dihedrals and the Saxon−Woods potential was used to enhance conformational sampling about the O6–C6–C5–O5 dihedral angles.

For the Drude force field a non-equilibrium sampling MD (neMD) method^84^ was applied to enhance sampling. The neMD method involves a hybrid propagator algorithm that sequentially performs a standard MD simulation on the unperturbed potential energy surface that is followed by a ‘boosting’ phase that involves a time-dependent Hamiltonian based on a perturbed potential energy surface and then returns to the unperturbed potential energy surface. A Monte Carlo Metropolis criterion is applied to accept or reject the configuration generated from the boosting phase. In the neMD run during the Hybrid-Rest2 propagator boosting phase the solute-solvent interaction energy and the solute intramolecular energy were perturbed where the solute atoms were O5′, C2′, C1′ in the terminal sugar residue, O2, C2, C3 and C1 for M2M, O3, C3, C4 and C2 for M3M, O4, C4, C5 and C3 for M4M and O6, C5, O5 and C4 for M6M in the reducing end residue. This selection in conjunction with the Hybrid-Rest2 method mimics the effect of increasing temperature on the glycosidic linkages thereby leading to enhanced sampling.^85^ The Drude model is approximately 4-fold slower than the additive CHARMM force field including the computational overhead associated with the additional nonbond interactions, integration of the Drude particles and the use of a 1 fs integration timestep *versus* a 2 fs timestep with the additive force field. Four neMD were performed for 100 ns each yielding a total of 400 ns with coordinates saved every 5 ps for analysis that was based on the ground-state portion of the trajectories only. Convergence of the simulations was checked based on potentials of mean force of the glycosidic torsion angles *ϕ* and *ψ* calculated from the first and second halves of both the additive and Drude enhanced-sampling simulations. Results showed the shape and location of the minima to be highly similar in both cases indicating that adequate conformational sampling with both force fields had been obtained.

For analysis, the effective ^1^H,^1^H distances in the disaccharides were calculated from the MD simulations according to *r*_*ij*_ = 〈*r*_*ij*_^−6^〉^−1/6^, where the brackets indicate averaging over all frames of a trajectory, and from NMR experiment by applying the isolated spin–pair approximation *r*_*ij*_ = *r*_ref_(*σ*_ref_/*σ*_*ij*_)^1/6^, where the reference distance *r*_ref_ is an average over the MD trajectory and *σ* is the proton–proton cross-relaxation rate constant between directly interacting proton pairs.^86^

## Results and discussions

### Translational and rotational motion

The Stokes–Einstein equation relates the translational diffusion constant (*D*_t_) to the radius of a sphere and subsequent application of the Debye–Stokes expression allows the rotational correlation time (*τ*_c_) to be obtained.^87^ Previous measurements by NMR spectroscopy at 298 K of translational diffusion coefficients for disaccharides with the same molecular formula as the mannobioses investigated herein, *viz.*, for α-d-Man*p*-(1→2)-α-d-Glc*p*-OMe and α-d-Man*p*-(1→2)-β-d-Glc*p*-OMe prepared at 100 mM solutions in D_2_O resulted in *D*_t_ = 3.6 × 10^−10^ m^2^ s^−1^ and *D*_t_ = 3.7 × 10^−10^ m^2^ s^−1^, respectively.^88^ Translational diffusion coefficients were determined for the four mannobioses, where a highly dilute solution <5 mM was used for M2M whereas for the other mannobioses the solute concentration was ∼60 mM in D_2_O. The highest value was observed for M2M with a *D*_t_ = 4.09 × 10^−10^ m^2^ s^−1^; for M3M*D*_t_ = 3.78 × 10^−10^ m^2^ s^−1^, for M4M*D*_t_ = 4.02 × 10^−10^ m^2^ s^−1^, and for M6M*D*_t_ = 3.94 × 10^−10^ m^2^ s^−1^. The six structurally similar disaccharides all show *D*_t_ ≈ 4 × 10^−10^ m^2^ s^−1^, but are, *inter alia*, influenced by the differences in solute concentration and consequently the effective solvent viscosity of the saccharide–solvent mixture. Furthermore, the disaccharide β-l-Fuc*p*-(1→6)-α-d-Glc*p*-OMe analyzed by Pendrill *et al.*^89^ showed at a 50 mM concentration in D_2_O and a temperature of 298 K a *D*_t_ = 4.00 × 10^−10^ m^2^ s^−1^, consistent with the data for the mannobioses.

The rotational correlation time of disaccharides M3M and M4M at 310 K in D_2_O, for which 1D ^1^H,^1^H-NOESY and ^1^H,^1^H-T-ROESY data were obtained at 600 MHz herein (*vide infra*), were calculated as previously described^90,91^ resulting in *τ*_c_ = 111 ps and *τ*_c_ = 93 ps, respectively. These correlation times are slightly shorter than that for β-l-Fuc*p*-(1→6)-α-d-Glc*p*-OMe at 303 K, which had *τ*_c_ = 116 ps.^89^ Effective correlation times (*τ*_eff_) can be obtained from the ratio between proton–proton cross-relaxation rates obtained by NOESY and T-ROESY experiments (*vide infra*) as previously described.^37,89^ For β-d-Glc*p*NAc-(1→6)-α-d-Man*p*-OMe at a 40 mM concentration in D_2_O and a temperature of 310 K^37^ as well as for β-l-Fuc*p*-(1→6)-α-d-Glc*p*-OMe the procedure based on cross-relaxation rates resulted in *τ*_eff_ ≈ 120 ps, for the latter in excellent agreement with the rotational correlation time derived from the translational diffusion measurements. Applying the same methodology to M3M and M4M for different proton-pairs revealed effective correlation times shorter than those obtained based on translational diffusion measurements indicating internal motion and dynamics not revealed by the diffusion data. It may be noted that the glycosidic linkage substitution occurs for (1→6)-linked disaccharides at a primary carbon atom (C6), while for M3M and M4M it takes place at a secondary carbon atom (C3 and C4, respectively). The geometrical arrangement in the latter case facilitates local or specific dynamics, in particular at the *ψ* torsion angle, which in α-l-Rha*p*-(1→2)-α-l-Rha*p*-OMe (essentially the mirror image of M2M) has a bimodal distribution.^92,93^

### NMR spectroscopy

**Fig. 2:**
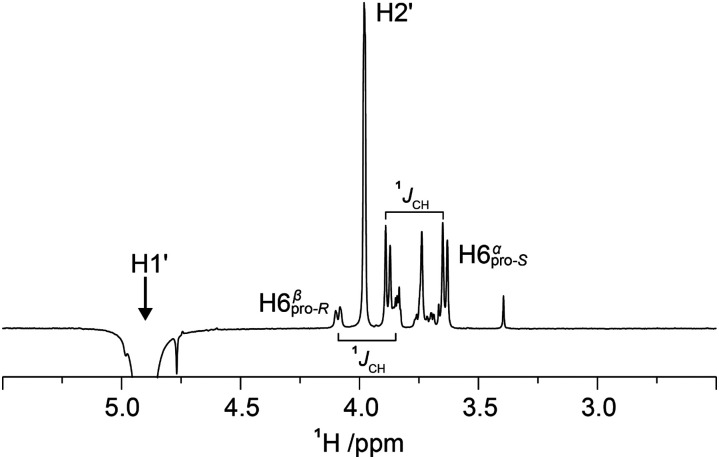
Selected spectral region of a 1D ^1^H,^1^H-NOESY NMR spectrum of M6M-*c* recorded at 298 K on a 600 MHz spectrometer with selective excitation of the anomeric proton H1' and a mixing time of 400 ms. The H6 resonances of the reducing end residue are split by ^1^*J*_CH_ due to the presence of the site-specific ^13^C label at carbon 6. Integration of NOE peaks to H6 protons was carried out for the α spin-state of H6_pro-*S*_ (*δ*_H_ 3.64) and the β spin-state of H6_pro-*R*_ (*δ*_H_ 4.09) and were multiplied by a factor of two for subsequent conformational analysis.

**Fig. 3:**
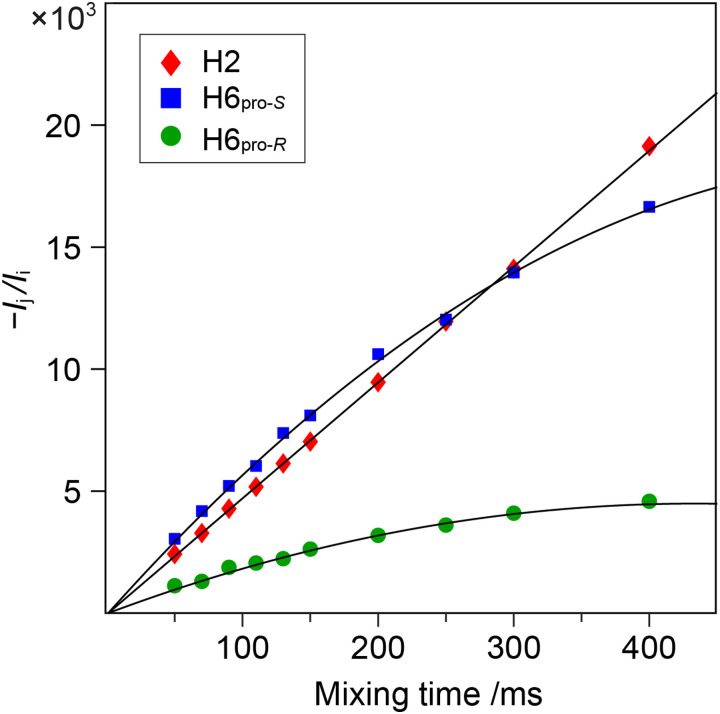
Plots of –*I*_j_(*τ*_m_)/*I*_i_(*τ*_m_) *versus τ*_m_ for the 1D ^1^H,^1^H-NOESY NMR experiments at 600 MHz with selective excitation applied to H1' of the disaccharide M6M-*c*. Cross-relaxation rates were obtained by the PANIC approach analyzed to first order for H2' (red-filled diamond) and to second order for H6_*pro*-S_ (blue-filled square) and H6_*pro*-R_ (green-filled circle).

1D ^1^H,^1^H-NOESY and ^1^H,^1^H-T-ROESY NMR experiments were carried out to obtain ^1^H,^1^H cross-relaxation rates from which inter-proton distances were derived. Selective excitation at the ^1^H NMR resonance frequency of the anomeric proton in the terminal mannosyl residue of M3M and of M4M employing an array of mixing times facilitated buildup curves that were analyzed by the PANIC approach,^94^ from which cross-relaxation rates were obtained (Table 1). In M6M the ^1^H NMR chemical shifts of H2′ and H6_pro-*S*_ overlapped severely^95^ precluding an intra-residue dipolar interaction from H1′ (selectively excited) to be determined separately from that of the inter-residue interaction across the glycosidic linkage. This limitation was circumvented by using α-d-Man*p*-(1→6)-α-d-[6-^13^C]Man*p*-OMe (M6M-*c*)^37^ for which the ^13^C-isotope labeling results in splitting of the H6 resonances due to the one-bond ^1^*J*_C6,H6_ couplings. From 1D ^1^H,^1^H-NOESY (Fig. 2) and ^1^H,^1^H-T-ROESY NMR spectra transglycosidic ^1^H,^1^H interactions could be detected and quantified using buildup curves that were analyzed by the PANIC approach,^94^ which in general facilitates a first order analysis at longer mixing times (*τ*_mix_) than for classical NOE buildup curves.^96^ Whereas the intra-residue H1′,H2′ NOE showed a linear relationship for *τ*_mix_ all the way up to 400 ms those of H1′,H6_pro-*R*_ and H1′,H6_pro-*S*_ deviated from linearity already at shorter mixing times (Fig. 3) attributed to different auto-relaxation rate constants^97^ for the anomeric proton H1′ and the two H6 methylene protons at the ^13^C nucleus of the isotope labeled α-(1→6)-linked disaccharide. The corresponding ^1^H,^1^H cross-relaxation differences for ^1^H,^12^C–^1^H,^12^C pairs on one hand and ^1^H,^12^C-^1^H,^13^C pairs on the other were previously observed for β-d-Glc*p*NAc-(1→6)-α-d-[6-^13^C]Man*p*-OMe^37^ when analyzed by a slightly different approach devised by Dixon *et al.*^98^ The intra-residue cross-relaxation rate (*σ*) was determined from the relationship −*I*_j_(*τ*_mix_)/*I*_i_(*τ*_mix_) = *σ*_ij_*τ*_mix_ where *I*_j_ is the area of the peak of interest and *I*_i_ is the area of the inverted peak, *i.e.*, *σ* is given by the slope of the H1′,H2′ interaction. For the inter-residue interactions in M6M-*c* a second order term was required^89,97^ and the initial rate was obtained by fitting the buildup curves of H1′,H6_pro-*R*_ and H1′,H6_pro-*S*_ to a second order polynomial. The determined cross-relaxation rates from the ^1^H,^1^H-NOESY and ^1^H,^1^H-T-ROESY NMR experiments are compiled in Table 1 together with derived distances obtained by the isolated spin-pair approximation in conjunction with MD simulations.

**Table 1:** NMR ^1^H,^1^H cross-relaxation rates × 10^2^ in s^−1^ at 600 MHz and interproton distances in Å from the enhanced-sampling MD simulations using additive C36 and Drude force fields for the four methyl mannobiosides

Compd	Proton pair	Expt	*σ*	*r*_HH_ (Expt)^f^	*r*_HH_ (C36)^g^	*r*_HH_ (Expt)^f^	*r*_HH_ (Drude)^g^
M2M^a^	H1′–H1	T-ROE	1.8	3.13	3.12	3.15	3.20
	H1′–H2	T-ROE	15.2	2.19	2.24	2.21	2.18
	H1–H5′	T-ROE	6.2	2.54	2.49	2.56	2.42
	H1′–H2′	T-ROE	7.4	2.47^h^	2.47	2.49^h^	2.49
M3M	H1′–H3	NOE^b^	9.24	2.27	2.27	2.29	2.19
	H1′–H2′	NOE^b^	5.62	2.47^h^	2.47	2.49^h^	2.49
	H1′–H3	NOE^c^	9.26	2.28	2.27	2.30	2.19
	H1′–H2′	NOE^c^	5.77	2.47^h^	2.47	2.49^h^	2.49
	H1′–H3	T-ROE	10.9	2.26	2.27	2.28	2.19
	H1′–H2′	T-ROE	6.40	2.47^h^	2.47	2.49^h^	2.49
M4M	H1′–H4	NOE^b^	8.62	2.25	2.26	2.28	2.23
	H1′–H2′	NOE^b^	4.80	2.48^h^	2.48	2.51^h^	2.51
	H1′–H4	NOE^c^	8.55	2.25	2.26	2.28	2.23
	H1′–H2′	NOE^c^	4.81	2.48^h^	2.48	2.51^h^	2.51
	H1′–H4	T-ROE	9.23	2.25	2.26	2.27	2.23
	H1′–H2′	T-ROE	5.10	2.48^h^	2.48	2.51^h^	2.51
M6M^d^	H1′–H6_pro-*R*_	NOE	2.08	2.85	2.88	2.86	2.62
	H1′–H6_pro-*S*_	NOE	6.22	2.37	2.36	2.38	2.32
	H1′–H2′	NOE	4.78	2.48^h^	2.48	2.49^h^	2.49
	H1′–H6_pro-*R*_	T-ROE^e^	2.97	2.78	2.88	2.79	2.62
	H1′–H6_pro-*S*_	T-ROE^e^	8.17	2.35	2.36	2.36	2.32
	H1′–H2′	T-ROE^e^	5.93	2.48^h^	2.48	2.49^h^	2.49

a T-ROE on M2M-l-Ser.^99^

b 1D DPFGSE-NOESY experiments.

c 1D SPFGSE-NOESY experiments with zero-quantum suppression filter.^48^

d Measured on M6M-*c*.

e Measured at a ^1^H NMR frequency of 500 MHz.

f Interproton distances calculated according to *r*_*ij*_ = *r*_ref_(*σ*_ref_/*σ*_*ij*_)^1/6^.

g Effective proton–proton distances averaged over MD trajectories according to *r* = 〈*r*^−6^〉^−1/6^.

h Reference distance from MD simulation.

**Fig. 4:**
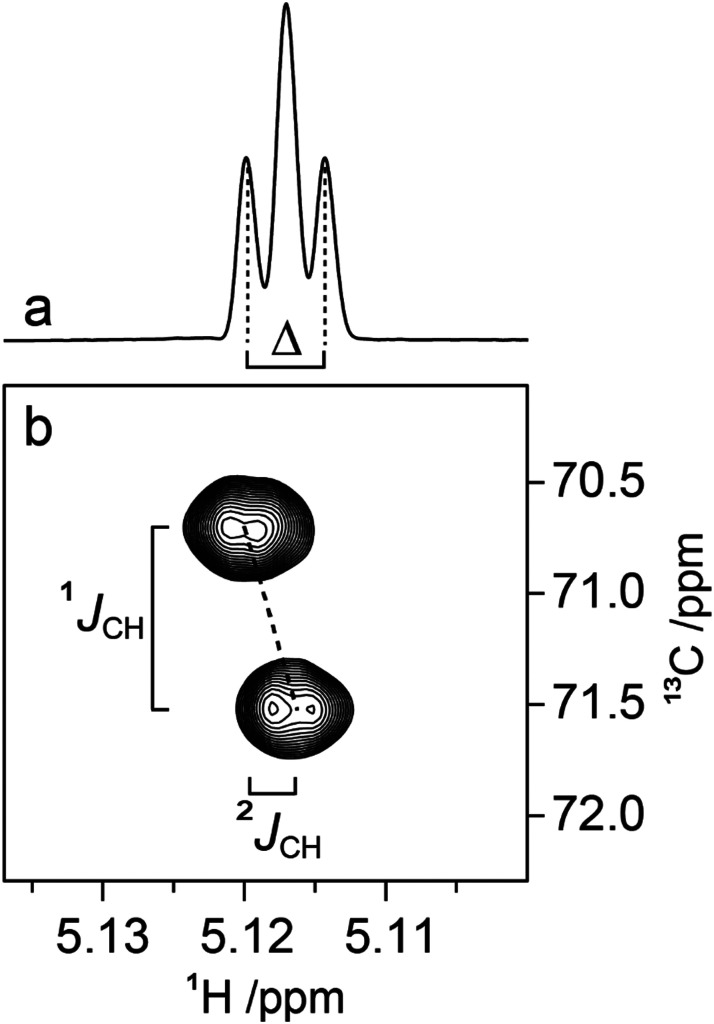
(a) ^1^H NMR resonance of the anomeric proton of the terminal mannosyl residue in M4M-*c*_2_ (α spin-state) where *Δ* = 4.2 Hz, ^3^*J*_H1′,H2′_ = 1.9 Hz; (b) selected region from the ^1^H,^13^C-HSQC-HECADE NMR spectrum at 700 MHz of M4M-*c*_2_ demonstrating the tilting of the cross-peak and the resulting *J*_C2′,H1′_ coupling constant of −2.3 Hz measured in the *F*_2_ dimension.

**Fig. 5:**
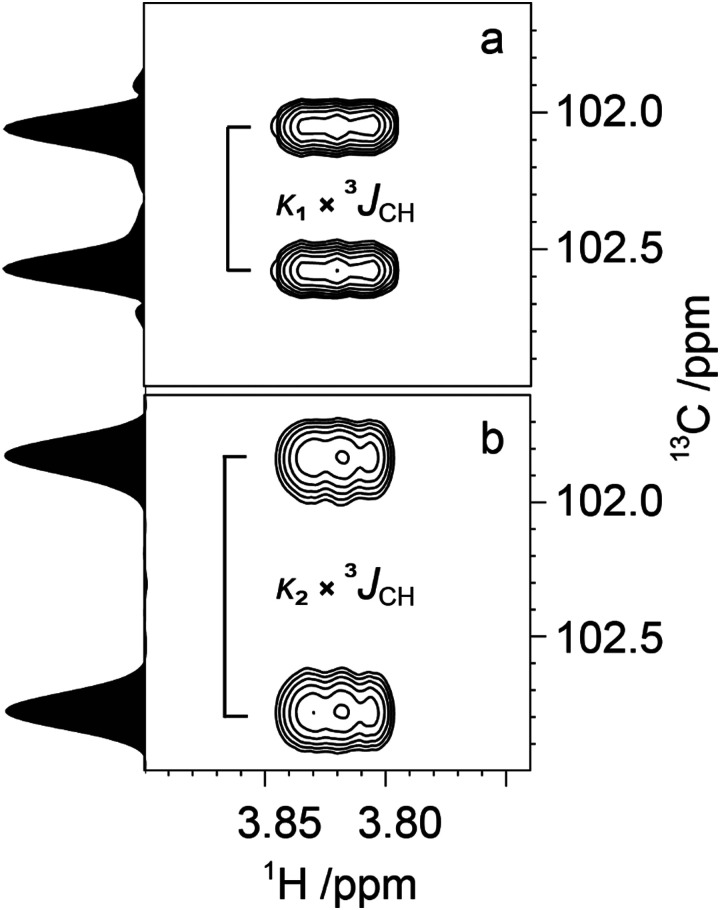
Spectral region of the C1'–H4 cross-peak of M4M-*c*_2_ from a ^13^C-CT-J-HMBC experiment and corresponding projections using a scaling factor *κ*_1_ = 18.7 (a) and *κ*_2_ = 33.0 (b).

Determination of ^3^*J*_CH_ scalar coupling constants in M4M was carried out by a 1DLR NMR experiment^50,51^ in conjunction with the *J* doubling procedure,^52^ as well as by a J-HMBC experiment^49^ resulting in heteronuclear transglycosidic coupling constants in full agreement with each other (Table 2). Subsequent NMR experiments to obtain conformationally dependent *J* couplings utilized M4M-*c*_2_ from which ^1^*J*_C1′,H1′_ and ^*n*^*J*_CC_ were determined from a 1D ^13^C{^1^H} NMR spectrum; ^3^*J*_C2′,C4_ and ^3^*J*_C1′,C4_ were assigned in analogy with M2M and M3M^58^ in which the former is larger and the latter is smaller and has a negative sign.^100^ Interestingly, the anomeric proton of the terminal mannosyl residue in M4M-*c*_2_ showed a *ddd* multiplet structure where the *dd* sub-multiplet from the ^1^*J*_C1′,H1′_ splitting showed an outer-peak separation of *Δ* = 4.2 Hz (Fig. 4a). From M4M we have ^3^*J*_H1′,H2′_ = 1.9 Hz and consequently |^2^*J*_C2′,H1′_| = 2.3 Hz. The sign of the latter coupling constant was determined from a ^1^H,^13^C-HSQC-HECADE NMR spectrum (Fig. 4b) in which the tilt was opposite to that of the ^2^*J*_C2′,H3′_ coupling that in mannose has a positive sign.^101^ Furthermore, the tilt of both the ^1^*J*_C1′,H1′_ and ^1^*J*_C2′,H2′_ correlation peaks in comparison to those of the ^*n*^*J*_CH_ doublet components indicates the relative signs of the coupling constants over one bond *vs.* multiple bonds, a cross-peak pattern observed in HSQC-HECADE spectra as previously shown by Koźmiński and Nanz.^55^ The magnitude of the heteronuclear two-bond coupling determined from the 2D NMR spectrum of M4M-*c*_2_ was in excellent agreement with that from the ^1^H NMR spectrum; thus ^2^*J*_C2′,H1′_ = −2.3 Hz in M4M as determined from the doubly ^13^C-labled isotopologue, similar to the corresponding two-bond heteronuclear coupling constant in α-d-Man*p*-(1→3)-β-d-Man*p*-OMe for which ^2^*J*_C2′,H1′_ = −1.9 Hz.^58^ The ^1^H,^13^C-HSQC-HECADE NMR experiment was previously applied to α-l-[2′-^13^C]Rha*p*-(1→2)-α-l-Rha*p*-OMe, which resulted in ^2^*J*_C2′,H1′_ = −2.2 Hz,^102^ also with a negative sign and of the same magnitude for the terminal sugar residue that has the *manno*-configuration. The ^3^*J*_C1′,H4_ coupling constant could furthermore be obtained using M4M-*c*_2_ but this required a 2D NMR experiment, which we refer to as ^13^C-J-HMBC,^53^ in which the interference from one-bond ^13^C,^13^C scalar coupling is suppressed by incorporating either a selective inversion pulse or a constant-time element in the pulse sequence. The latter version was used herein and two different scaling factors (*κ*) were employed resulting in 2D NMR spectra with correlations of interest (Fig. 5). The one-dimensional projections were used to obtain the peak separation (Δ*F*_1_) and subsequent calculation using the relationship *J*_CH_ = Δ*F*_1_/*κ* facilitated determination of the ^3^*J*_C1′,C4_ coupling constant, which was in excellent agreement with data from the natural abundance sample M4M (Table 2).

**Table 2:** Experimental transglycosidic ^*n*^*J*_CH_ and ^*n*^*J*_CC_ NMR coupling constants in Hz and those calculated from the enhanced-sampling MD simulations using additive C36 and Drude force fields for the four methyl mannobiosides. For the α-(1→6)-linked mannose disaccharide ^2^*J*_H5,C6_ and ^3^*J*_H5,H6_ related to the *ω* torsion angle are also given

Compd	Torsion angle	Atom pair	^*n*^ *J*_expt_	^*n*^ *J*_calc_ (C36)	^*n*^ *J*_calc_ (Drude)
M2M	*ϕ*_H_	H1′–C2	4.1^a^	3.5	3.5
	*ψ*_H_	C1′–H2	4.6^a^	4.6	5.0
	*ϕ*_C2′_	C2′–C2	3.6^b^	4.1	3.9
	*ϕ*_C1′_	C1′–C2	−1.8^b^	−2.5	−2.5
	*ψ*_C3_	C1′–C3	2.0^b^	2.8	2.2
M3M	*ϕ*_H_	H1′–C3	3.8^c^	3.2	3.4
	*ψ*_H_	C1′–H3	5.0^c^	5.3	5.0
	*ϕ*_C2′_	C2′–C3	3.4^b^	4.1	3.9
	*ϕ*_C1′_	C1′–C3	−1.8^b^	−2.5	−2.4
	*ψ*_C3_	C1′–C4	1.4^b^	1.7	2.2
M4M	*ϕ*_H_	H1′–C4	4.2^d^, 4.2^e^	4.4	4.0
	*ϕ*_C2′_	C2′–C4	2.8^f^	3.5	3.4
	*ϕ*_C1′_	C1′–C4	−1.9^f^^g^	−2.3	−2.3
	*ϕ*_C2′H1′_	C2′–H1′	−2.3^h^	−2.2	−2.2
	*ϕ*_H1′_	H1′–C1′	171.8	169.9	169.9
	*ψ*_H_	C1′–H4	5.1^d^, 5.0^e^, 5.0^i^	4.8	4.6
	*ψ*_C3_	C1′–C3	0.5^f^^j^	0.9	1.0
	*ψ*_C5_	C1′–C5	2.1^f^^j^	3.1	3.1
M6M	*ϕ*_H_	H1′–C6	3.4^k^	2.5	3.5
	*ϕ*_C2′_	C2′–C6	3.5^l^	4.1	3.7
	*ψ*_R_	C1′–H6_pro-*R*_	2.7^k^	2.7	3.3
	*ψ*_S_	C1′–H6_pro-*S*_	2.5^k^	2.1	2.9
	ω_C6_	H5–C6	−1.7^l^	0.1	−0.1
	ω_R_	H5–H6_pro-*R*_	5.1^l^	4.8	4.3
	ω_S_	H5–H6_pro-*S*_	2.0^l^	1.8	1.9

a Säwén *et al.*^59^

b Zhang *et al.*^58^

c Pendrill *et al.*^35^

d 1DLR experiment (J-doubling 16 times).

e J-HMBC experiment.

f ^*n*^ *J*_CC_ determined on M4M-*c*_2_.

g Two-bond scalar coupling assumed to have a negative sign.

h Sign of two-bond scalar coupling determined by a ^1^H,^13^C-HSQC-HECADE NMR experiment on M4M-*c*_2_ in which ^2^*J*_C2′,H3′_ ≈ +1.1 Hz.

i ^13^C-CT-J-HMBC experiment on M4M-*c*_2_.

j Determined at an elevated temperature of 333 K.

k Patel *et al.*^71^

l Olsson *et al.*^37^

### Comparison between NMR experimentally derived and simulation computed data

**Fig. 6:**
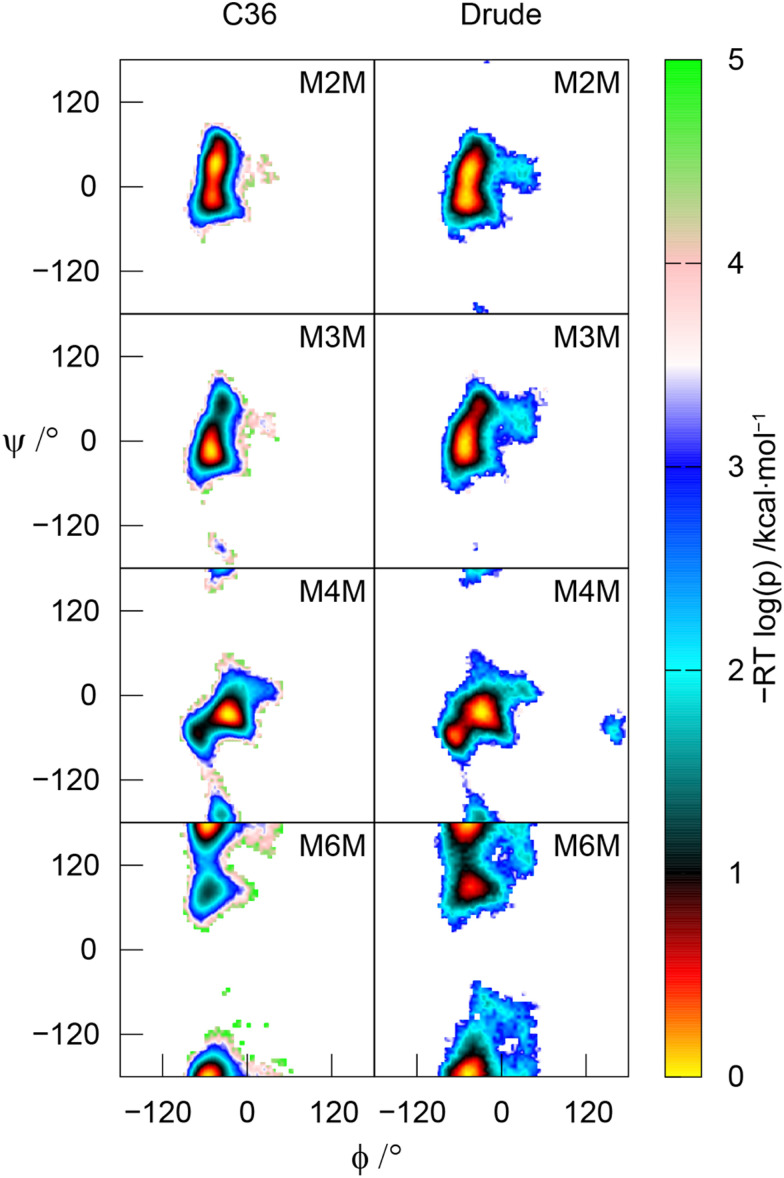
Potential of mean force (PMF) of the glycosidic torsion angles *ϕ* and *ψ* in the four α-linked mannopyranose disaccharides. Top to bottom: M2M, M3M, M4M and M6M with the additive C36 force field (left column) and the polarizable Drude force field (right column).

The MD simulations using both the CHARMM36 and Drude force fields were used to calculate effective inter-proton distances, which showed very good to excellent agreement to those derived from 1D ^1^H,^1^H-NOESY and ^1^H,^1^H-T-ROESY NMR experiments (Table 1). In a few cases the Drude model results in somewhat shorter effective distances than those observed employing the C36 force field, presumably due to the higher conformational flexibility of the disaccharides in the former case, as can be seen from the 2D potential of mean force (PMF) profiles along the glycosidic torsion angles *ϕ* and *ψ* (Fig. 6).

In comparing the experimental transglycosidic ^*n*^*J*_CH_ and ^*n*^*J*_CC_ NMR coupling constants (Table 2) to those calculated from the MD simulations using the pertinent Karplus-type relationships many agree well. However, the one-bond heteronuclear coupling constant ^1^*J*_C1′,H1′_ in M4M deviates, for which the experimental value of ^1^*J*_C1′,H1′_ = 171.8 Hz; a comparison to M2M having ^1^*J*_C1′,H1′_ = 171.2 Hz and M3M with ^1^*J*_C1′,H1′_ = 171.8 Hz^58^ shows that these one-bond coupling constants are comparable, indicating a similar conformational preference for the ϕ torsion angle in the three disaccharides. In a previous study on the pentasaccharide LNF-1^103,104^ a one-bond Karplus-type relationship for β-linked hexopyranosides facilitated the identification of the preferred exo-anomeric conformational region for the *ϕ* torsion angle, consistent with complementary experimental NMR data. The one-bond Karplus-type relationship for α-linked hexopyranosides, having a span of ∼8 Hz (eqn 1), was utilized in the analysis of the MD simulations that resulted in a deviation of the computed ^1^*J*_C1′,H1′_ value (Table 2) to the experimentally determined one for both the CHARMM36 and Drude FFs. The two-bond heteronuclear coupling constant ^2^*J*_C2′,H1′_ in M4M (Table 2), for which the span of the Karplus-type relationship (eqn 2) is ∼5 Hz, shows computed values from the MD simulations resulting in ^2^*J*_C2′,H1′_ = −2.2 Hz, consistent with a synperiplanar conformation at the *ϕ* torsion angle and in excellent agreement with the experimentally determined value for ^2^*J*_C2′,H1′_ = −2.3 Hz. The recently developed Karplus-type relationship developed for α-d-Man*p*-(1→3)-β-d-Man*p*-OMe,^57^ but herein applied to M4M is a significant improvement over the previously reported one,^105^ which differed by ∼1 Hz (less negative value) when calculated from the CHARMM-based MD simulations presented herein. Analysis of the *J* coupling data for the CHARMM36 and Drude force fields shows that the overall agreement is closely similar with an RMSD of 0.71 ± 0.02 Hz using all acquired data (Table 2), which decreases to an RMSD of 0.53 ± 0.01 Hz when the two heteronuclear coupling constants ^1^*J*_C1′,H1′_ in M4M (*vide supra*) and ^2^*J*_C6,H5_ in M6M (*vide infra*) were omitted from the data set.

The three-bond heteronuclear ^3^*J*_H1′,C*n*_ where *n* is the substitution position and homonuclear ^3^*J*_C2′,C*n*_ related to the *ϕ* torsion angle are generally somewhat underestimated and overestimated by the MD simulations, respectively, irrespective of the force field (except for M6M with the Drude force field for which excellent agreement is observed between experiment and simulation) suggesting that fine-tuning of Karplus-type relationships and/or force fields will alleviate these small deviations, *e.g.*, if the force field would be tuned in such a way that the *mean* position of the *ϕ* torsion angle is altered slightly, from the –synclinal region (−90° to −30°) to the synperiplanar region (−30° to +30°). The transglycosidic homonuclear ^2^*J*_C1′,C*n*_ coupling constants with a glycosidic linkage at a secondary carbon atom, *i.e.*, M2M – M4M, show from the MD simulations computed values for ^2^*J*_C1′,C*n*_ < −2 Hz whereas for the experimentally determined ones these ^2^*J*_C1′,C*n*_ > −2 Hz (Table 2). This may indicate that non-exo *ϕ* conformations^59,106^ are also present to some extent (*cf.* also Fig. 5 in Zhang *et al.*^58^). The conformational preferences at the *ϕ* torsion angle of mannobioses was recently investigated in a study by Meredith *et al.*^107^ in which experimental transglycosidic ^3^*J*_CH_ and homonuclear ^3^*J*_CC_ and ^2^*J*_CC_ NMR coupling constants were utilized in an analysis in which the population of *ϕ* is represented by a sum of wrapped normal distributions. The study revealed a discrepancy between, in particular, the circular standard deviation which was twice or three times as large in a unimodal description from experimental *J* data compared to that obtained by MD simulations using the GLYCAM06 force field. Furthermore, the mean position of *ϕ* from the MD simulations deviated from the one resulting from experimental data; by constraining the models to conformations consistent with the *ψ* distribution from experiment, agreement to GLYCAM06-derived mean *ϕ* torsions could be obtained. The study provided evidence that further force field development is warranted to obtain consistency between simulation and experiment.

The MD simulations reproduce *J* data related to the transglycosidic torsion angle *ψ* quite well but varies somewhat between the CHARMM36 and Drude force fields (Table 2). These results in conjunction with NOE-based data show that the PMF maps and a bimodal distribution at the *ψ* torsion angle for glycosidic linkages at a secondary carbon atom, besides an anti-*ψ* conformation that may contribute to a small extent in M4M, give a representative description of the conformational space spanned at the glycosidic linkage of the disaccharides. In the 4-*O*-substituted disaccharide the homonuclear ^3^*J*_C1′,C*n*±1_ coupling constants related to the *ψ* torsion angle show a small coupling to C3 and a large one to C5 (Table 2) revealing a preference toward the vicinity of a +synclinal conformation (+30° to +90°) for the torsion angle of the former coupling path whereas the antiperiplanar conformation is supported by the latter torsion along the coupling path; both force fields correctly describe these *J* interactions, although the latter ^3^*J*_C1′,C5_ is overestimated by the MD simulations. For M6M in which the glycosidic connectivity is present *via* a primary carbon atom and the *ψ* torsion angle is defined by C1′–O6–C6–C5 the major conformer has an antiperiplanar orientation and the minor conformation is observed at *ψ* ≈ +90° (Fig. 6); the ^3^*J*_C1′,H6_ coupling constants related to this *ψ* torsional angle are consistent with the conformational space sampled at the α-(1→6)-linkage in M6M. The presence of the second conformational state at the *ψ* torsion angle is in accordance with that predicted for β-l-Fuc*p*-(1→6)-α-d-Glc*p*-OMe,^89^*i.e.*, the geometrical arrangement at the glycosidic linkage for the hexoses with β-l- and α-d-configuration substituting hexoses with *gluco*/*manno*-configurations is similar.

**Fig. 7:**
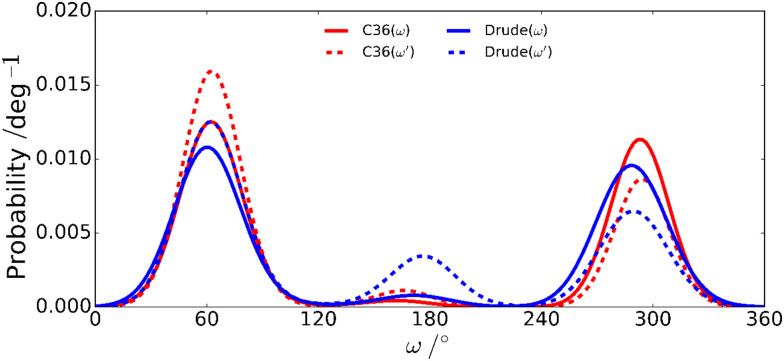
Rotameric distributions of the *ω* torsion angles in M4M for the additive C36 and the polarizable Drude force fields.

The rotamer populations of *ω* torsion angles in hexoses with *gluco*/*manno*-configuration have a *gt*:*gg* ratio of ∼1 : 1 with only a small amount of the *tg* conformer.^108–111^ The CHARMM36 and Drude force fields differ for the mannobioses in that the *ω*′ torsion of the terminal residue has the *gt* conformational state as the predominant one for CHARMM36 whereas for the Drude force field the *tg* conformation is significantly populated (Table 3). The population distribution for the *ω* torsion angles in M4M derived from NMR ^3^*J*_H5,H6_ coupling constants showed relative populations for the three states *gt* : *gg* : *tg* of 5 : 4 : 1, with only minute differences due to temperature, a phenomenon also observed in a study of hydroxymethyl rotamer populations of cello-oligosaccharides as a function of temperature over a large temperature range.^112^ Notably, for M6M with an α-(1→6)-linkage both force fields reproduce the change in rotamer population favoring for *ω* instead the *gg* conformational state as the major one, in agreement with results derived from NMR data, and consistent with rotamer populations of *ω* in favoring the *gg* conformational state in α-d-Glc*p*-(1→6)-α-d-Glc*p*-OMe^71^ and β-l-Fuc*p*-(1→6)-α-d-Glc*p*-OMe.^89^ However, the computed ^2^*J*_C6,H5_ coupling constant, for which the Karplus curve spans ∼8 Hz (eqn 11), deviated from the experimental value (Table 2) for both force fields used. Furthermore, the herein calculated value of ^2^*J*_C6,H5_ was closely similar to that obtained from a previous MD simulation of M6M using the CHARMM36 force field.^71^ Like for the *ϕ*/*ψ* maps describing the conformational space at the glycosidic linkage of the mannobioses a broader population distribution is exhibited at *ω* torsions for the Drude force field compared to the classical CHARMM36 force field for the studied disaccharides (Fig. 7 and Fig. S1–S3, ESI†).

**Table 3:** Rotamer populations (%) for *ω* torsion angles of four methyl mannobiosides from MD simulations at 310 K and derived from experimental ^1^H NMR spin–spin coupling constants

	*ω*′ C36	*ω* C36	*ω*′ Drude	*ω* Drude	*ω*′ NMR	*ω* NMR
M2M						
*gt*	70	57	55	45		
*gg*	25	38	31	39		
*tg*	5	5	14	16		
M3M						
*gt*	67	60	51	48		
*gg*	28	35	34	36		
*tg*	5	5	15	16		
M4M^a^^b^						
*gt*	63	53	54	50	51 (49)	52 (50)
*gg*	32	45	30	46	42 (42)	41 (41)
*tg*	5	2	16	4	7 (9)	7 (9)
M6M^c^						
*gt*	56	∼41	47	33		45
*gg*	39	59	36	59		49
*tg*	5	< 1	17	8		6

a 297 K from Daikoku *et al.*^34^ with H5′,H6′_pro-*R*_ = 5.7 Hz, H5′,H6′_pro-*S*_ = 2.1 Hz, H5,H6_pro-*R*_ = 5.8 Hz and H5,H6_pro-*S*_ = 2.1 Hz.

b (In parenthesis) 343 K from Rönnols *et al.*^113^ with H5′,H6′_pro-*R*_ = 5.57, H5′,H6′_pro-*S*_ = 2.24 Hz, H5,H6_pro-*R*_ = 5.72 Hz and H5,H6_pro-*S*_ = 2.29 Hz.

c 310 K from Olsson *et al.*^37^ with H5,H6_pro-*R*_ = 5.12 Hz and H5,H6_pro-*S*_ = 1.96 Hz.

A number of studies based on NMR spectroscopy experiments and molecular modeling on mannose-based oligosaccharides have been reported over the years, with the results generally consistent with those obtained in the present study. For example, compounds M2M, M3M and M6M were studied by Palivec *et al.*^114^ using Raman spectroscopy combined with molecular modeling using the GLYCAM06 force field.^115^ Free energy surfaces and sampling during the MD simulations of the α-(1→2)- and α-(1→3)-linkages were similar to the present observations with respect to the highest probability region; however, a wider region of conformational space was sampled in comparison to both the C36 additive and Drude FF, though closer to that obtained with the Drude model with sampling observed in the vicinity of approximately *ϕ* = 30° and *ψ* = 30° in both studies. In the α-(1→6)-linked species six local minima on the *ϕ*/*ψ* free energy surface were investigated using, *inter alia*, biased MD simulations in which the system is restrained to a specific conformational region, since the *ω* torsion angle was rather confined with a sharp profile having O6–C6–C5–H5 ≈ −48° corresponding to the *gt* conformational state, in contrast to significant populations of both the *gt* and *gg* conformational states in M6M employing the CHARMM-based FF herein. Using the GLYCAM06 FF similar sampling along the torsion angle *ψ* was obtained for the glycosidic linkage with more sampling along the torsion angle *ϕ* including in the vicinity of 180°, not seen in the present study. In a study of a trimannoside corresponding to the oligosaccharide branching region in N-glycoproteins ^1^H,^1^H-NOESY NMR experiments and acquired trans-glycosidic three-bond ^1^H,^13^C NMR coupling constants were utilized in conjunction with MD simulations with explicit water molecules as solvent to obtain information on its three-dimensional shape and flexibility.^116^ Four major conformations arouse from two-state populations at each of the α-(1→3)- and α-(1→6)-linkages; moreover, two additional conformational states were identified for the latter glycosidic linkage. A study of α-(1→2)-linked *O*-mannobiose using MM3* with a Generalized Born/Solvent accessible term to model the aqueous environment identified two major conformational regions,^117^ which overlap with the ones observed in the present study, though the populations of conformations were reversed. For *C*-glycosyl analogues of di- and tri-mannosides the conformational space is significantly increased.^117–119^ Moreover, results from even earlier studies combining NMR analysis with modeling on various oligomannosides largely produced a picture of conformations similar to those observed in the present study, although differences in the modeling results were often obtained primarily based on the use of gas phase calculations in which the solute molecule was devoid of surrounding solvent present under experimental conditions resulting in more approximate modeling methods at that time.^36,120–124^

### Molecular dipole analyses using additive and Drude polarizable force fields

**Fig. 8:**
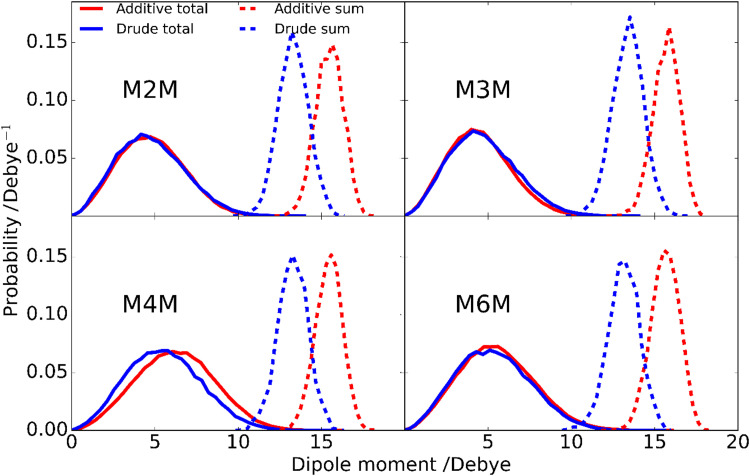
Probability distributions of the total dipole moments of the disaccharides (lines) and of the sum of the dipole moments of hydroxyl groups (dashed) for additive C36 (red) and Drude polarizable (blue) FFs. Hydroxyl dipole moments include contributions from the OH group and covalently linked CH_*n*_ atoms, as well as lone pairs with the Drude polarizable FF as their charges sum to zero.

**Fig. 9:**
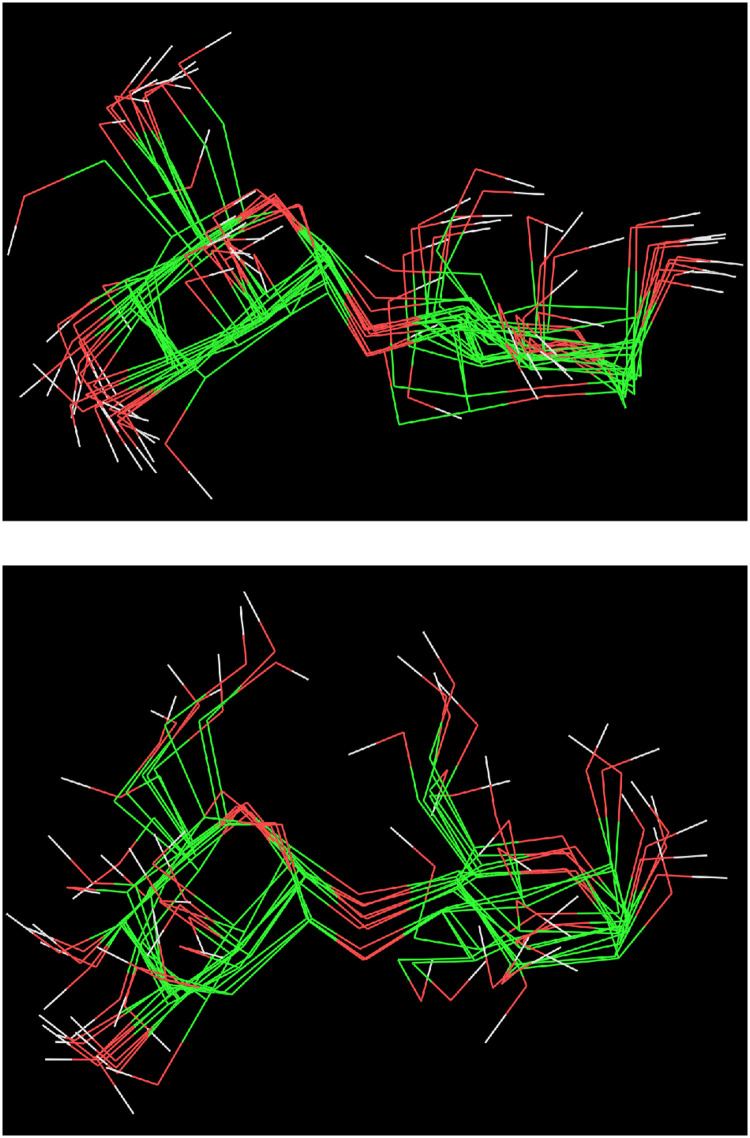
Selected conformations of the M4M disaccharide from the simulations with the Drude polarizable force field resulting in an average in dipole moments of ∼14 Debye (top) or less than 0.1 Debye (bottom). Of the hydrogen atoms, only those of the hydroxyl groups are shown for clarity.

Analysis of the enhanced sampling MD simulations using the Drude polarizable and CHARMM36 additive force fields beyond the comparisons of the experimental and computed conformational properties discussed above were undertaken to obtain additional details on molecular structure. In particular, the impact of the explicit inclusion of electronic polarizability in the Drude FF was evaluated through comparisons with the additive CHARMM36 force field through analysis of dipole moments. The inclusion of the explicit treatment of electronic polarizability in the Drude FF may contribute to the differences in the range of conformational sampling observed in the free energy profiles (Fig. 6). Accordingly, the dipole moment distributions of the disaccharides from the additive and Drude simulations were analyzed, both with respect to the total disaccharides and for the individual hydroxyl groups. Dipole moment calculations of the hydroxyl groups included the covalently-linked carbon and associated hydrogen atoms as the sum of the partial charges on this collection of atoms (H–O–C–H_*n*_), and lone pairs in the case of the Drude force field, equals zero, as required for the dipole moment to be spatially invariant. The dipole moment distributions of the total disaccharides and the sum of their constituent hydroxyl groups (Fig. 8) reveal that the distributions for the total disaccharides are quite similar for the CHARMM36 and Drude FFs, ranging from zero to up to 14 Debye. This result was somewhat unexpected given the lack of explicit electronic polarizability in CHARMM36, where changes in dipole moments only occur due to changes in geometry. However, large differences are observed in the distributions of the sum of the hydroxyl dipole moments, indicating difference in the underlying contributions to the dipole moments in the two FFs. To understand the similarity of the total dipole moment distributions, selected conformations of the disaccharides were obtained in which the dipole moments were <0.1 or ∼14 Debye. When large values of the overall dipole moment occur, the hydroxyl groups are aligned (Fig. 9) such that the summation of their individual contributions yield large overall dipole moments, even with for additive force field. In contrast, when the hydroxyl groups are not aligned (Fig. 9) the individual dipoles of the hydroxyl groups cancel each other, leading to the small overall dipole moments.

**Fig. 10:**
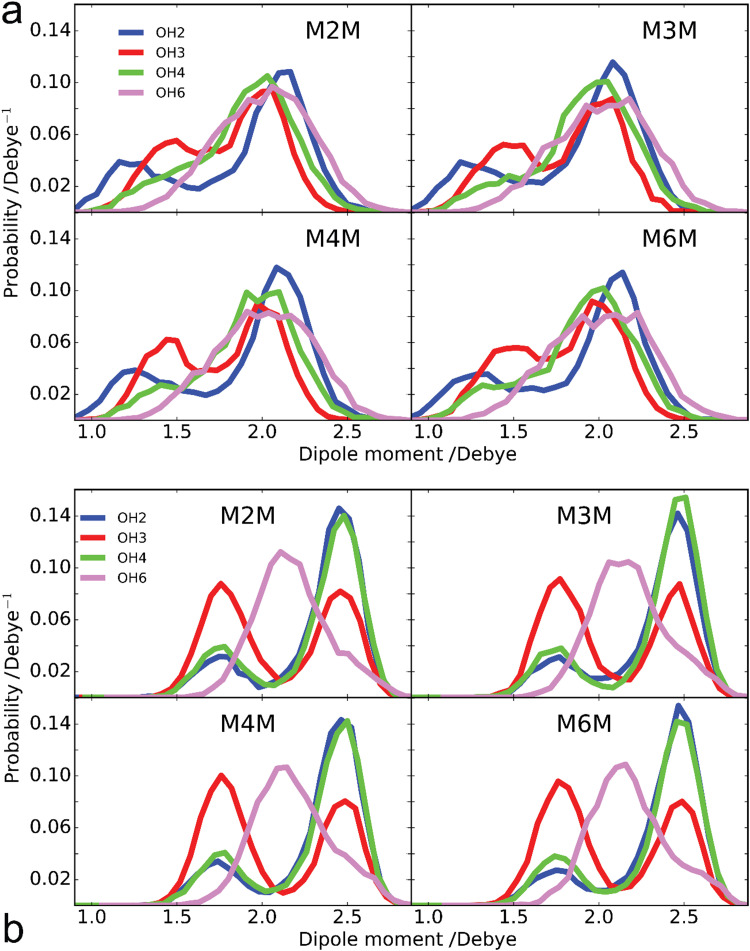
Dipole moment probability distributions of hydroxyl groups at positions 2 (blue), 3 (red), 4 (green) and 6 (purple) from the (a) Drude polarizable and (b) additive C36 FF simulations. Results are summed over both monosaccharides in the four disaccharides as the individual distributions are similar (Fig. S4 and S5, ESI†). Hydroxyl dipole moments include contributions from the OH group and covalently linked CH_*n*_ atoms, as well as lone pairs with the Drude polarizable FF as their charges sum to zero.

While the overall dipole moments of the disaccharides are similar for CHARMM36 and the Drude force fields the hydroxyl dipole moments are systematically larger in the additive model (Fig. 8). This is expected due to the overestimation of the empirical dipole moments *versus* gas phase experimental or QM values in the additive force field to account for the impact of condensed phase environment.^125^ However, it would be anticipated that the Drude dipole moments may sample large values in certain orientations or due to interactions with the environment. Alternatively, in situations where adjacent hydroxyl groups are aligned, the dipole–dipole interactions would dampen the magnitude of the individual dipole moments. To better understand the observed behavior, the dipole moments of the individual hydroxyl groups were analyzed. For the different disaccharides the behavior of the dipole distributions was similar (Fig. S4 and S5, ESI†); the combined dipole moment distributions over both monosaccharides in the four disaccharides are presented in Fig. 10 for the two force fields used. In general, with the Drude FF the dipole moment distributions are broader, the peaks of the distributions are typically shifted to lower values and smaller values of the dipole moments are being sampled (Fig. S4, ESI†) as compared to the additive CHARMM36 FF (Fig. S5, ESI†). The overall shapes of the distributions are generally similar for the two force fields employed, though OH4 groups peak at ∼2 Debye when the Drude FF is used whereas also a well-defined second peak occurs with CHARMM36; for OH3 groups the magnitude of the two maxima are more similar with CHARMM36. While the maxima with the Drude FF are generally at lower values, the distributions do sample large values similar to those occurring with the additive FF indicating that the Drude hydroxyls can assume larger dipole moments in certain orientations though the sum over all the hydroxyl groups is systematically lower than with CHARMM36 (Fig. 8). This appears to be due to the damping effect discussed above that can occur with the polarizable model leading to dipole moments of <1 Debye. For OH6, only a single maximum is observed and the location is similar in the two force fields, though the Drude distribution is wider. This single maximum, and similar values in the two FFs, is likely due to the more solvent exposed nature of the primary alcohol *versus* the secondary alcohols (OH2–OH4). Thus, while the overall distributions of the dipole moments of the disaccharides employing the Drude and CHARMM36 FFs are similar there are differences in those of the individual hydroxyl groups with wider distributions occurring from the Drude FF, which may contribute to the broader range of conformations being sampled by the polarizable FF as seen in the 2D PMFs for glycosidic torsion angles *ϕ* and *ψ* (Fig. 6).

**Fig. 11:**
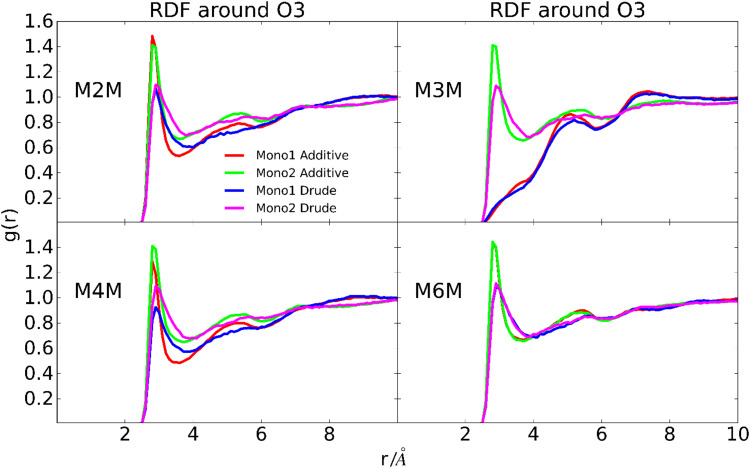
Radial distribution functions of water oxygen atoms around O3 of the hydroxyl group for the four disaccharides. Results are shown for the individual monosaccharides for the additive C36 and Drude polarizable force fields as indicated by the label in the figure. Monosaccharide 1 and 2 correspond to the reducing and terminal end residues of the disaccharides, respectively.

To further investigate possible contributions to the wider distribution of conformations in the Drude FF (Fig. 6) radial distribution functions (RDF) of water with the oxygen atoms of the hydroxyl groups, O5 and O1 sites were calculated. The location of the peaks in the RDFs, including both the primary and secondary peaks, are similar for the two force fields (Fig. 11 depicts the RDF of OH3 and Fig. S6 displays the other RDFs, ESI†). However, with the Drude FF the magnitudes of the primary peaks are lower and they are systematically wider than those with the additive CHARMM36 force field. This is evident for all the hydroxyl groups while the differences are less pronounced for the O5 and glycosidic bond oxygen atoms (Fig. S6, ESI†). The lower and wider peaks with the Drude FF in combination with the wider distributions in the dipole moment distributions indicate more variability in the hydroxyl–water interactions in the polarizable force field. Overall, these results show that while the overall disaccharide dipole moments for the additive and polarizable simulations are similar, differences in the dipoles of the individual hydroxyl groups are present and this contributes to less well-defined interactions of the hydroxyl groups with water for the Drude FF. This difference in the interactions with water is suggested to contribute to the wider range of conformations being sampled by the Drude polarizable *versus* the additive CHARMM36 force field.

## Conclusions

Conformational analysis was carried out on a series of disaccharides containing terminal α-d-Man*p* residues linked to α-d-Man*p*, thereby representing the four regioisomers that all are present in nature. ^13^C isotope labeling of oligosaccharides facilitated not only heteronuclear and homonuclear scalar coupling constants to be obtained and used *via* Karplus-type relationships together with MD simulations but also resolved spectral overlap in 1D ^1^H,^1^H-NOESY and 1D ^1^H,^1^H-T-ROESY NMR experiments by judicious choice of ^1^H NMR spectrometer frequency in relation to ^1^*J*_C6,H6_ scalar coupling of the ^13^C isotopologue in the α-(1→6)-linked disaccharide. The doubly ^13^C-isotope labeled α-(1→4)-linked disaccharide made it possible to acquire transglycosidic coupling constants used in the conformational analysis and application of the ^13^C-J-HMBC NMR experiment in which a one-bond ^13^C,^13^C scalar coupling was suppressed resulted in ^3^*J*_C1′,H4_ in excellent agreement with the scalar coupling determined from the natural abundance sample. That there is a quite close resemblance between the CHARMM36 and Drude force fields as analyzed by inter-residue proton–proton distances and NMR scalar coupling constants across glycosidic linkages, with both models in good agreement with the experimental data, gives credibility to the more recently developed Drude force field. Notably, sampling of a wider range of conformations, differences in the distribution of dipole moments and altered interactions between the hydroxyl groups and water in the two force fields indicate underlying differences in atomic contributions to the calculated NMR observables in the additive *versus* polarizable models. Thus, the present study represents an important step forward in obtaining a more detailed understanding of the conformational properties of mannose saccharides that builds upon four decades of combing experimental methods with molecular modeling. Furthermore, future applications to complex structures or assemblies such as in studies of carbohydrate-protein interactions^126–128^ may be aided by the improved description of the physical forces dictating the conformational properties obtained with the Drude polarizable force field model thereby representing molecular interactions in a more realistic way.

## Conflicts of interest

ADM Jr. is co-founder and CSO of SilcsBio LLC; for the other authors there are no conflicts to declare.

## Supplementary Material

CP-025-D2CP05203B-s001
