# Supplementary material for: Glycosidic α-linked mannopyranose disaccharides: an NMR spectroscopy and molecular dynamics simulation study employing additive and Drude polarizable force fields
Source: Phys Chem Chem Phys. 2022 Dec 21;25(4):3042–60. doi: 10.1039/d2cp05203b (PMC9890503; doi:10.1039/d2cp05203b)
Supplement: CP-025-D2CP05203B-s001 [file CP-025-D2CP05203B-s001.pdf]

Electronic Supplementary Material (ESI) for Physical Chemistry Chemical Physics.
This journal is © the Owner Societies 2022

# Glycosidic α-linked mannopyranose disaccharides: An NMR spectroscopy and molecular dynamics simulation study employing additive and Drude polarizable force fields

Alessandro Ruda,‡[a] Asaminew H. Aytenfisu,‡[b] Thibault Angles d'Ortoli,[a] Alexander D. MacKerell, Jr.*[b] and Göran Widmalm*[a]

[a] Department of Organic Chemistry, Arrhenius Laboratory, Stockholm University, S-106 91 Stockholm, Sweden

[b] Department of Pharmaceutical Sciences, School of Pharmacy, University of Maryland, Baltimore, Maryland 21201

‡A.R. and A.H.A. contributed equally to this work.

*Corresponding authors: alex@outerbanks.umaryland.edu and goran.widmalm@su.se

## Supporting information

### Table of contents

Rotameric distributions of the ω torsion angles page S2 – S4

Individual dipole moment distributions of hydroxyl groups page S5 – S6

Radial distribution functions of water oxygen and sugar oxygen atoms page S7 – S9

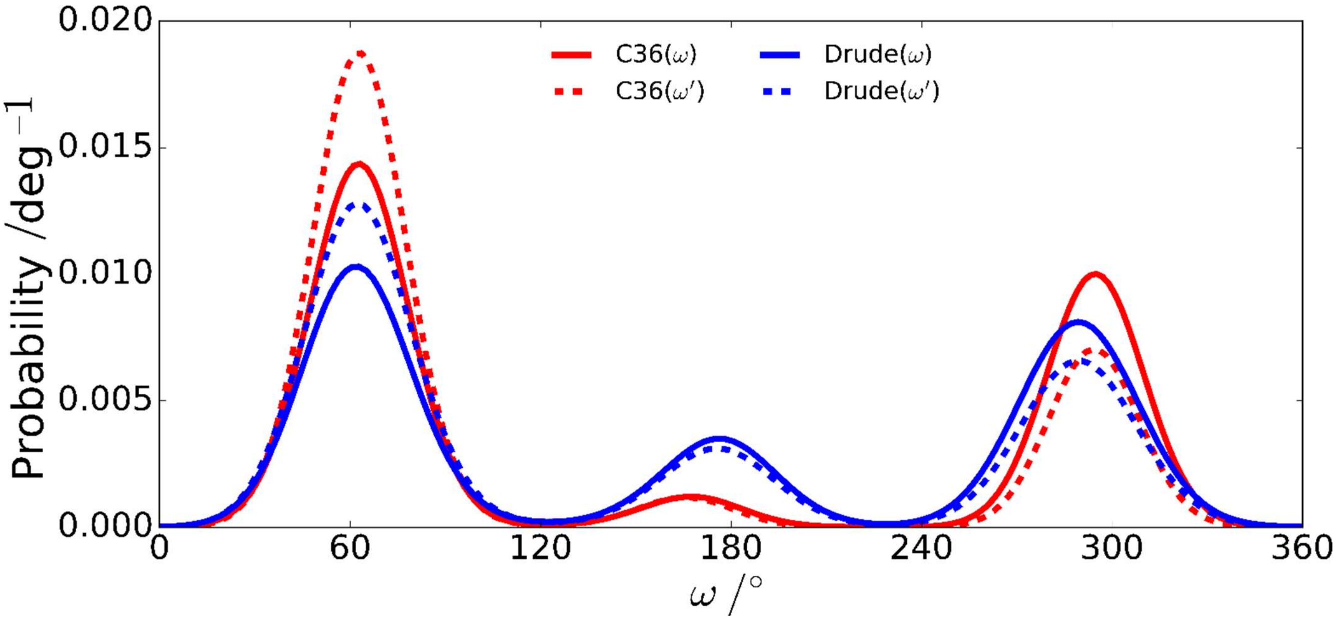


Figure S1. Rotameric distributions of the ω torsion angles in **M2M** for the additive C36 and the polarizable Drude force fields.

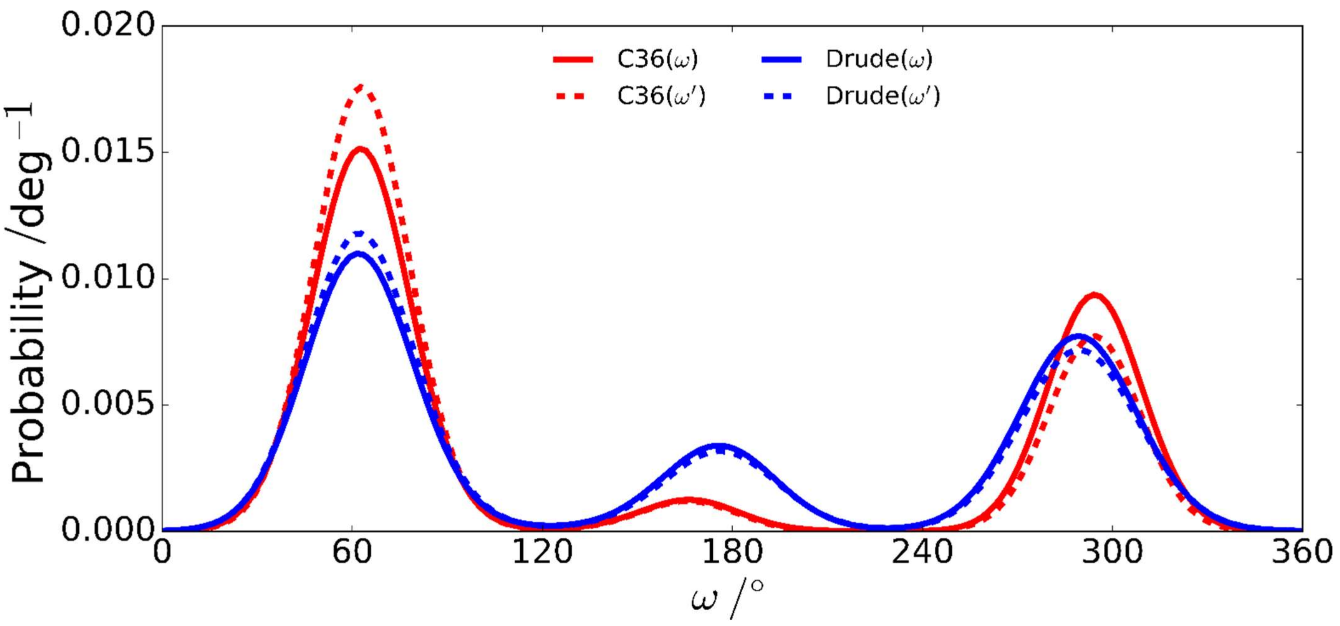


Figure S2. Rotameric distributions of the ω torsion angles in **M3M** for the additive C36 and the polarizable Drude force fields.

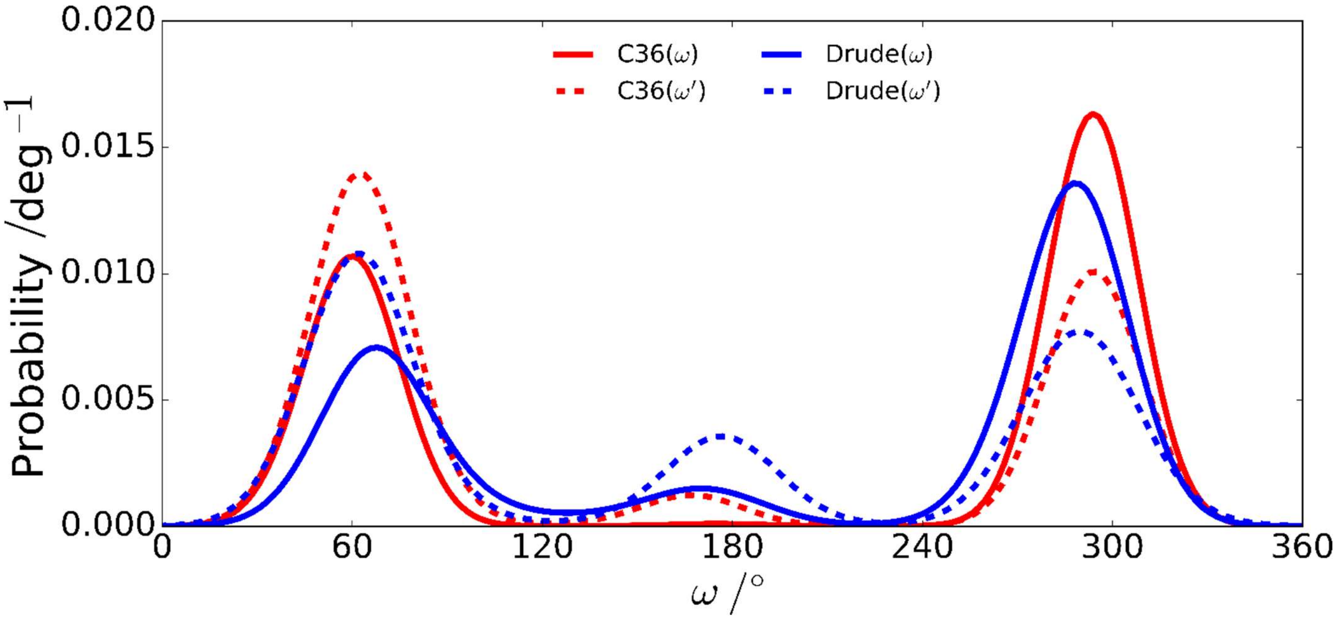


Figure S3. Rotameric distributions of the ω torsion angles in **M6M** for the additive C36 and the polarizable Drude force fields.

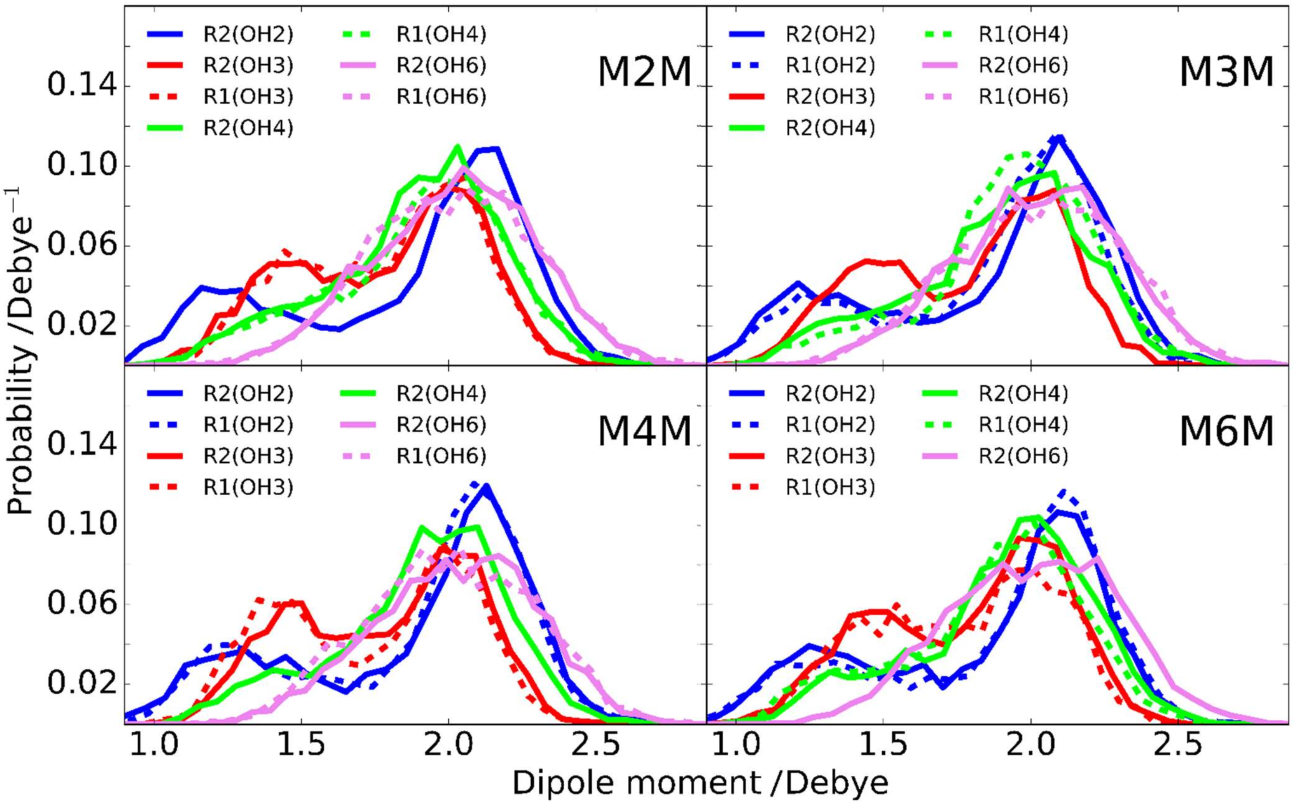


Figure S4. Individual dipole moment distributions of the hydroxyl groups of the α-linked mannopyranose disaccharides from the Drude simulations for the for the reducing end residue (R1, solid line) and the terminal residue (R2, dashed line). Results are shown for hydroxyl groups at positions 2 (blue), 3 (red), 4 (green) and 6 (purple). Hydroxyl dipole moments include contributions from the OH group, covalently linked $CH_n$ atoms, and lone pairs as their charges sum to zero. Note that each panel is devoid of a dashed line, since the oxygen atom of the missing hydroxyl group is part of the pertinent glycosidic linkage.

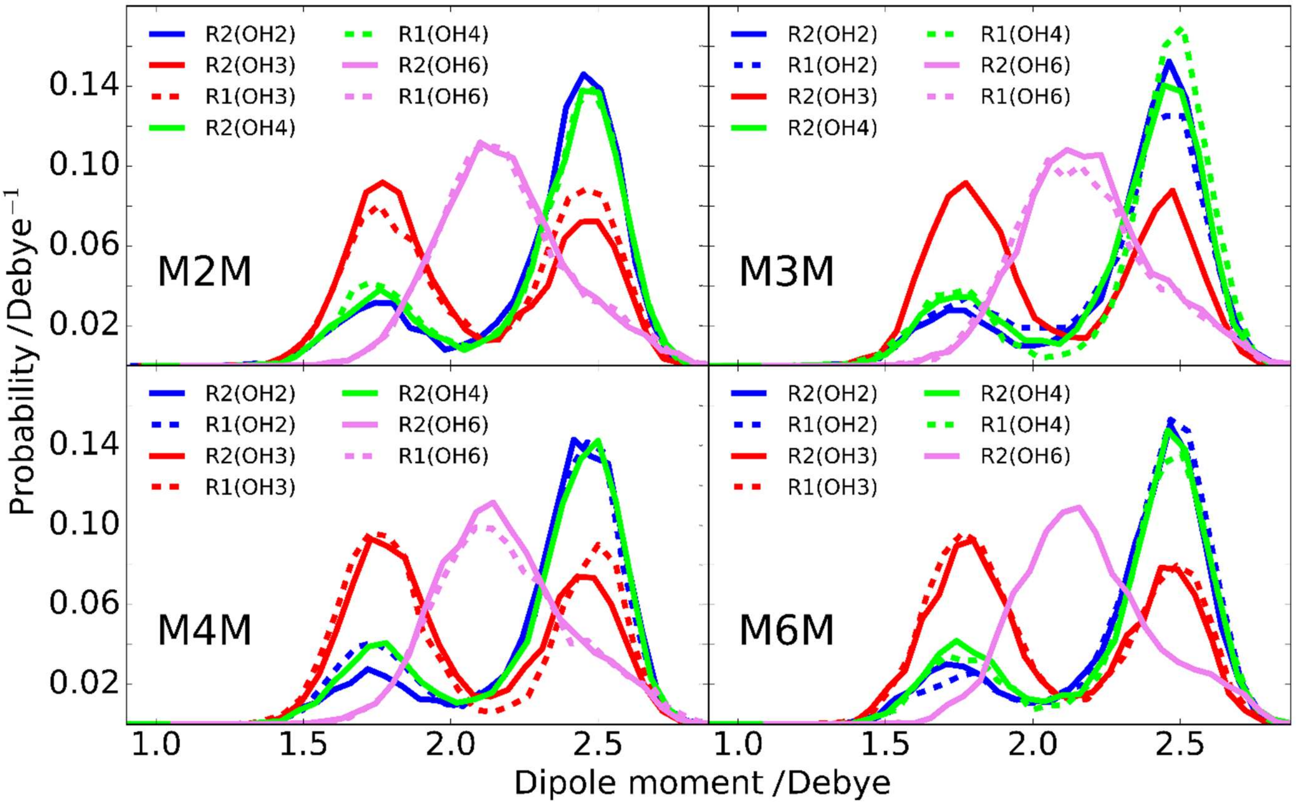


Figure S5. Individual dipole moment distributions of the hydroxyl groups of the α-linked mannopyranose disaccharides from the Additive C36 simulations for the reducing end residue (R1, solid line) and the terminal residue (R2, dashed line). Results are shown for hydroxyl groups at positions 2 (blue), 3 (red), 4 (green) and 6 (purple). Hydroxyl dipole moments include contributions from the OH group and covalently linked $CH_n$ atoms as their charges sum to zero. Note that each panel is devoid of a dashed line, since the oxygen atom of the missing hydroxyl group is part of the pertinent glycosidic linkage.

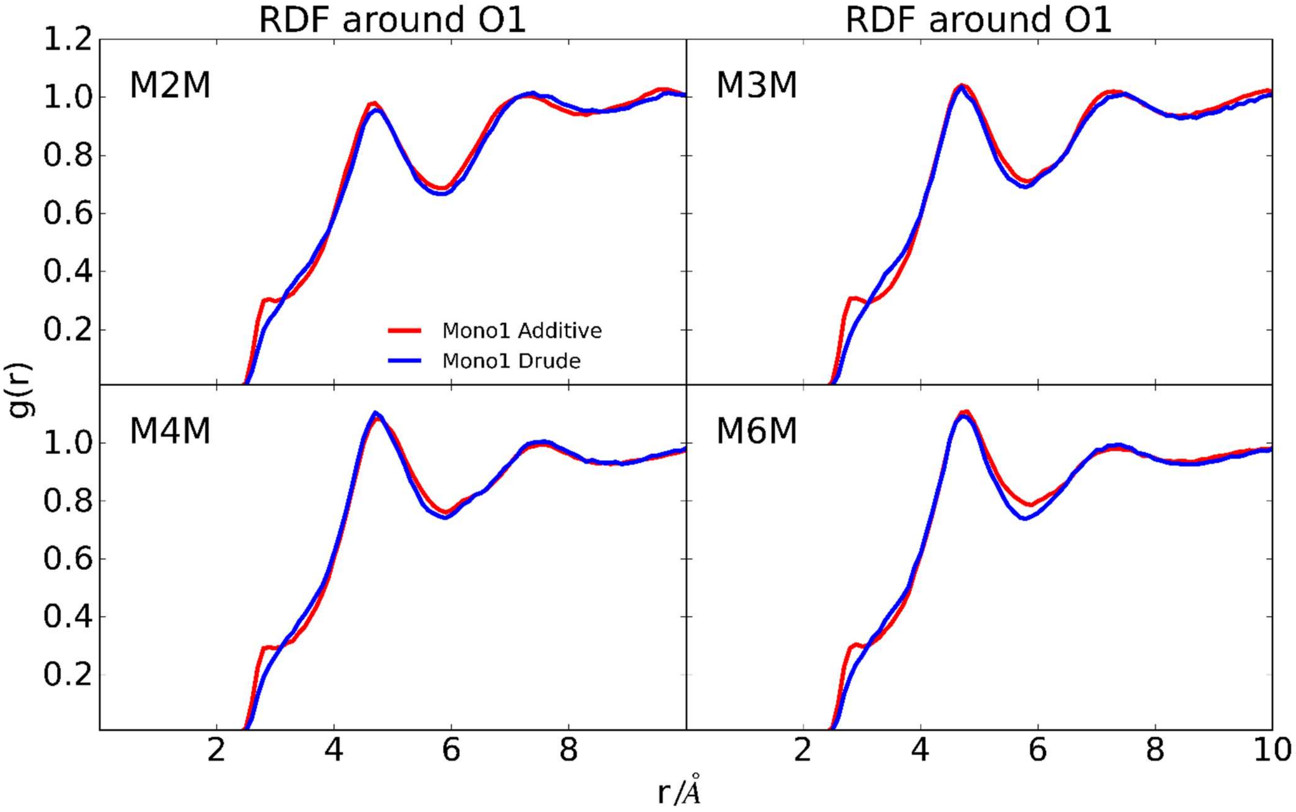
RDF around O1
RDF around O1
M2M
M3M
M4M
M6M
Mono1 Additive
Mono1 Drude
g(r)
r/Å

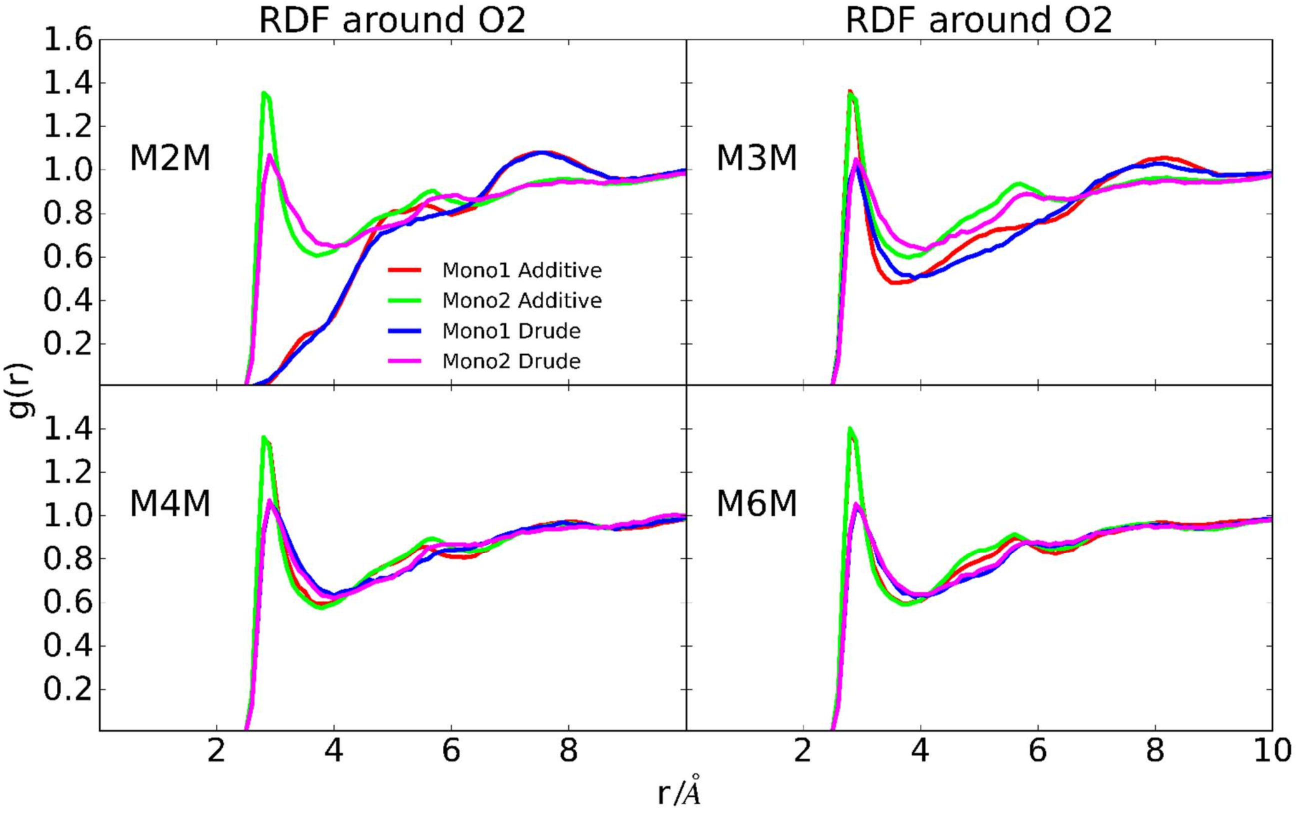
RDF around O2
RDF around O2
M2M
M3M
M4M
M6M
Mono1 Additive
Mono2 Additive
Mono1 Drude
Mono2 Drude
g(r)
r/Å

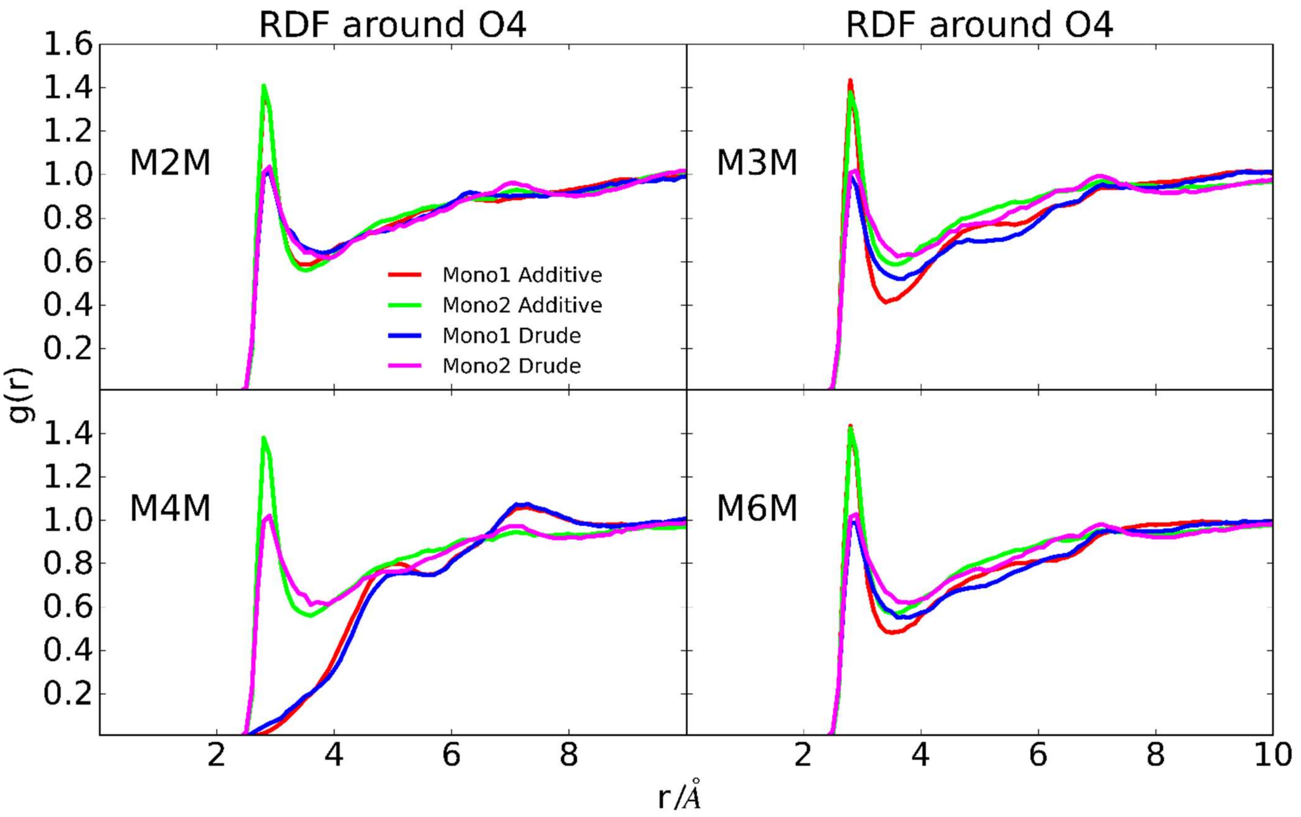
RDF around O4
RDF around O4
M2M
M3M
M4M
M6M
Mono1 Additive
Mono2 Additive
Mono1 Drude
Mono2 Drude
g(r)
r/Å

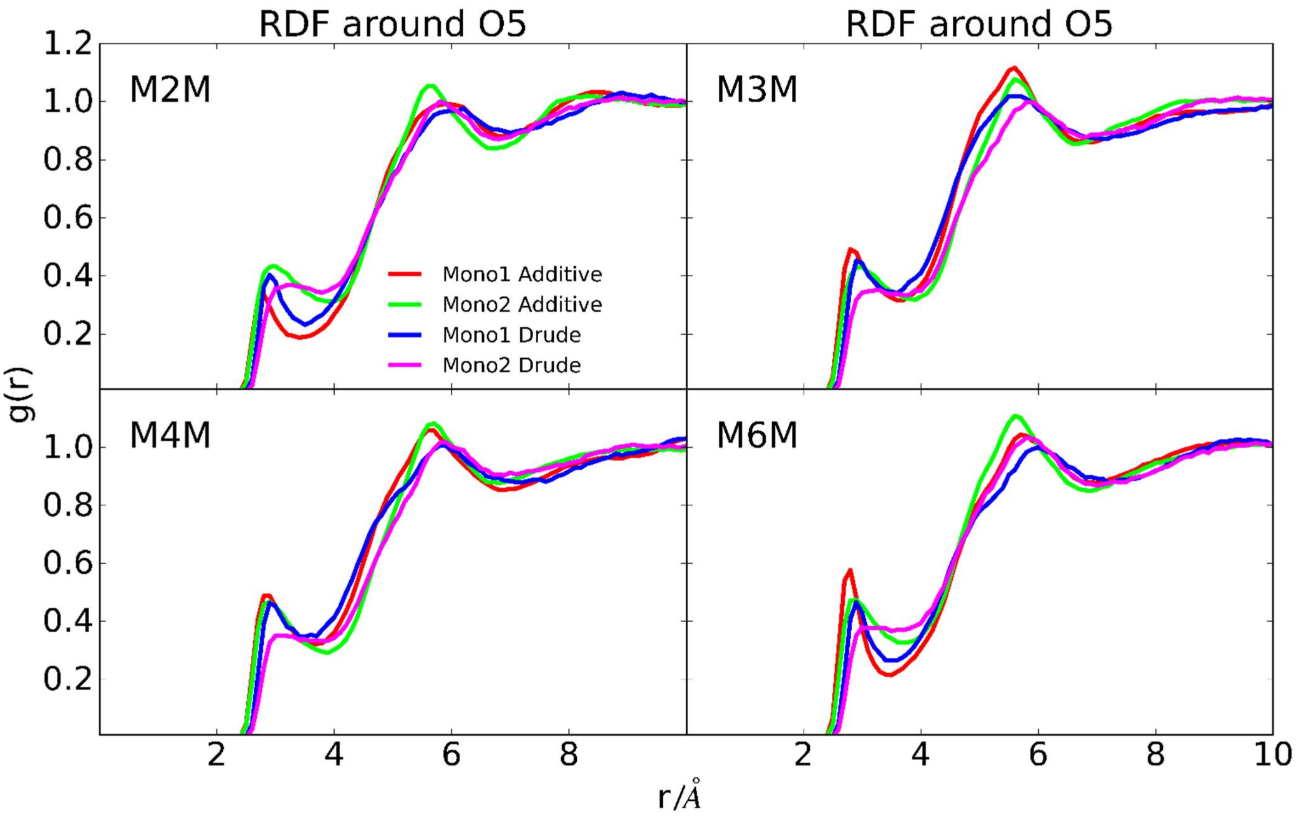
RDF around O5
RDF around O5
M2M
M3M
M4M
M6M
Mono1 Additive
Mono2 Additive
Mono1 Drude
Mono2 Drude
g(r)
r/Å

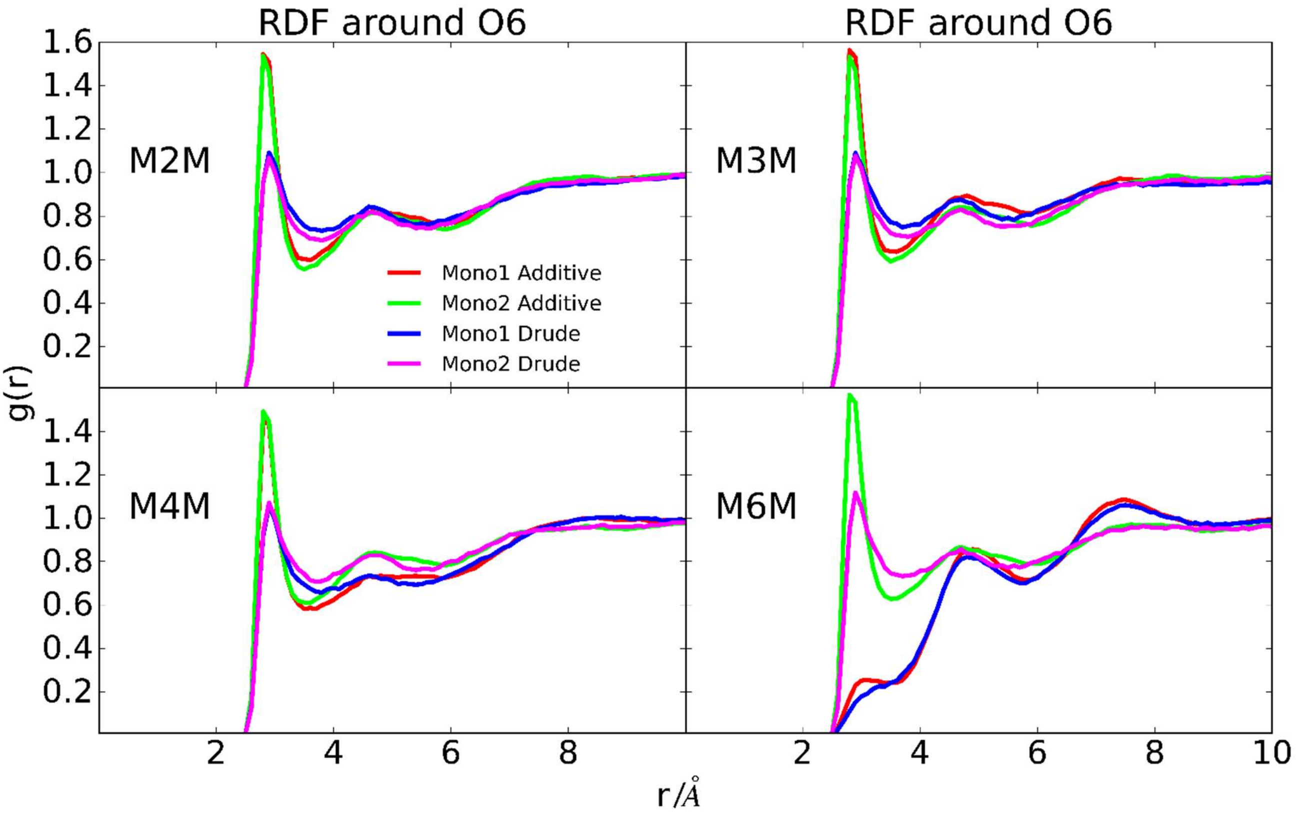


Figure S6. Radial distribution functions of water oxygen around the sugar oxygen atoms of the four disaccharides. Top to bottom panels depict RDFs for O1, O2, O4, O5 and O6. Monosaccharide 1 and 2 correspond to the reducing and terminal end residues of the disaccharides, respectively, for the additive C36 and Drude force fields as indicated by the labels the figure.
